# Osteoblast-Specific *Krm2* Overexpression and *Lrp5* Deficiency Have Different Effects on Fracture Healing in Mice

**DOI:** 10.1371/journal.pone.0103250

**Published:** 2014-07-25

**Authors:** Astrid Liedert, Viktoria Röntgen, Thorsten Schinke, Peggy Benisch, Regina Ebert, Franz Jakob, Ludger Klein-Hitpass, Jochen K. Lennerz, Michael Amling, Anita Ignatius

**Affiliations:** 1 Institute of Orthopaedic Research and Biomechanics, Center of Musculoskeletal Research, University of Ulm, Ulm, Germany; 2 Department of Osteology and Biomechanics, University Medical Center Hamburg-Eppendorf, Hamburg, Germany; 3 Orthopaedic Center for Musculoskeletal Research, University of Würzburg, Würzburg, Germany; 4 Institute of Cell Biology, University of Duisburg-Essen, Duisburg, Germany; 5 Institute of Pathology, University of Ulm, Ulm, Germany; University of Ulm, Germany

## Abstract

The canonical Wnt/β-catenin pathway plays a key role in the regulation of bone remodeling in mice and humans. Two transmembrane proteins that are involved in decreasing the activity of this pathway by binding to extracellular antagonists, such as Dickkopf 1 (Dkk1), are the low-density lipoprotein receptor related protein 5 *(Lrp5*) and Kremen 2 (*Krm2*). *Lrp 5* deficiency (*Lrp5^−/−^*) as well as osteoblast-specific overexpression of Krm2 in mice (*Col1a1-Krm2*) result in severe osteoporosis occurring at young age. In this study, we analyzed the influence of Lrp5 deficiency and osteoblast-specific overexpression of Krm2 on fracture healing in mice using flexible and semi-rigid fracture fixation. We demonstrated that fracture healing was highly impaired in both mouse genotypes, but that impairment was more severe in *Col1a1-Krm2* than in *Lrp5^−/−^* mice and particularly evident in mice in which the more flexible fixation was used. Bone formation was more reduced in *Col1a1-Krm2* than in *Lrp5^−/−^* mice, whereas osteoclast number was similarly increased in both genotypes in comparison with wild-type mice. Using microarray analysis we identified reduced expression of genes mainly involved in osteogenesis that seemed to be responsible for the observed stronger impairment of healing in *Col1a1-Krm2* mice. In line with these findings, we detected decreased expression of sphingomyelin phosphodiesterase 3 (Smpd3) and less active β-catenin in the calli of *Col1a1-Krm2* mice. Since Krm2 seems to play a significant role in regulating bone formation during fracture healing, antagonizing KRM2 might be a therapeutic option to improve fracture healing under compromised conditions, such as osteoporosis.

## Introduction

The canonical Wnt/β-catenin pathway has been intensively investigated over the past decade because of its key role in the regulation of skeletal development and bone mass maintenance [1]. The remarkable finding that loss-of-function mutations in the Wnt coreceptor low-density lipoprotein receptor related protein 5 (LRP5) gene cause the osteoporosis-pseudoglioma syndrome (OPPG), a rare autosomal recessive disorder of severe juvenile osteoporosis and congenital blindness, and that gain-of-function mutations in this gene result in a high bone mass phenotype, provided first evidence for the considerable influence of Lrp5 signaling on bone remodeling [2], [3]. In addition, studies using transgenic mouse models are currently reflecting the high impact of Wnt signaling on bone mass regulation [4]–[6]. Thus, targeted disruption of Lrp5 in mice results in a low bone mass phenotype due to decreased osteoblast proliferation and function [7]. The osteoporotic phenotype and persistent eye vascularization recapitulates the human OPPG syndrome. Although most of the transgenic animal models that affect bone mass specifically target canonical Wnt signaling, there is increasing evidence that noncanonical Wnt signaling pathways, the Wnt-planar cell polarity (Wnt-PCP) and the Wnt-calcium (Ca2+) pathway, play a significant role in bone mass homeostasis. Thus, it has been demonstrated that there may be a crosstalk between these pathways and that some Wnts are able to activate more than one of these pathways in a receptor-dependent manner. [1], [5].

There is evidence that canonical Wnt signaling needs to be downregulated in mature osteoblasts to enable bone matrix mineralization [8]. Therefore, extracellular antagonists, including Dickkopf 1 (Dkk1), a member of a small family of secreted proteins, are upregulated during osteoblast differentiation [6], [8]. Dkk1 binds to both coreceptors, Lrp5/6 and with high affinity to the transmembrane proteins Kremen 1 or 2 (Krm1, Krm2), thereby forming a ternary complex that undergoes rapid endocytosis and removal of the Lrp coreceptors from the cell membrane, resulting in an inhibition of Wnt/β-catenin signaling [9]. Deleting both, Krm1 and additionally Krm2 expression in mice leads to an increase of bone volume, which was comparable to that observed in haploinsufficient Dkk1 (+/−) mice [10]. Osteoblast-specific overexpression of Krm2 in transgenic mice (*Col1a1-Krm2*) results in severe osteoporosis that is associated with an impaired osteoblast maturation and bone formation as well as increased bone resorption [6]. Consequently, these data demonstrate that Krm2, at least in mice, is an important regulator of bone remodeling by inhibiting bone formation while increasing bone resorption.

Various studies have already shown the crucial role of Wnt/β-catenin signaling in bone fracture repair [11]. Canonical Wnt signaling has been shown to be important in the early phase of fracture repair to allow differentiation of mesenchymal cells into either chondrocyte or osteoblast lineages [12]. β-Catenin mediated Tcf-dependent transcription is activated during both, chondrogenesis as well as osteogenesis in fracture healing and is downregulated in the later phase of osteogenesis, as osteoblasts mature to osteocytes. Using various transgenic mouse models it has been demonstrated that a precisely stage-specifically regulated canonical Wnt signaling is necessary in order to allow complete fracture healing [13]. Fracture healing was impaired in mice conditionally expressing β-catenin null alleles. These mice had a lack of bone and cartilage and showed immature mesenchymal cells at the fracture site. Fracture healing was also repressed in mice expressing the null allele specifically in osteoblasts. These observations are in line with previous studies demonstrating that activated canonical Wnt signaling can interfere with the differentiation of skeletal precursors into chondrocytes and osteoblasts [14], [15]. Canonical Wnt signaling has been shown to maintain mesenchymal stem cells in a less differentiated state during osteogenic differentiation. In contrast, in cells already committed to the osteoblast phenotype, activated β-catenin signaling promotes osteoblastic differentiation and enhances osteogenesis, whereas in osteoblast precursors lacking β-catenin osteoblast differentiation is inhibited and they develop into chondrocytes [16], [17].

It has been shown that suppression of Wnt pathway inhibitors [18] and activation of β-catenin signaling can enhance healing, whereas β-catenin pathway inhibitors can delay bone regeneration [19]. Based on all these findings, it is obvious that Wnt/β-catenin signaling activation during a defined time during fracture healing might be an excellent option for the improvement of fracture healing under compromised conditions, such as osteoporosis. However, clarifying the role of signaling molecules regulating this pathway in fracture healing is a prerequisite to expand the small number of anabolic drugs that are currently available and evaluated for bone regeneration.

Apart from molecular factors, the mechanical environment that is determined by the fixation stability has an important influence on the fracture healing outcome [20]. Thus, more rigid fracture stabilization promotes intramembraneous ossification, whereas more flexible fracture fixation increases endochondral bone formation. Previously, we established standardized models for investigating the impact of mechanical factors on bone healing in mice, allowing for the adjustment of flexible and semi-rigid conditions [21].

In the present study we examined the influence of Lrp5 deficiency and osteoblast-specific Krm2 overexpression on fracture healing in mice with flexible and semi-rigid fixation, respectively. Both mouse models are known to be associated with an osteoporotic phenotype due to impaired Wnt signaling. We hypothesized that these mouse models show a genotype-specific delayed healing compared to wild-type mice that is more evident in mice with the more flexible fracture fixation compared to mice with the semi-rigid fracture fixation.

## Material and Methods

### Animals

The experiments were performed according to international regulations for care and use of laboratory animals, and approved by the local ethical committee (Regierungspräsidium Tübingen, No. 906, Germany). *Col1a1-Krm2* transgenic mice (*C57BL/6* genetic background) were generated as previously described [6]. In brief, the ORF encoding the Dkk receptor Krm2 was placed under the control of an osteoblast-specific *Col1a1* promoter fragment. Schulze et al. performed RT-PCR to confirm the bone-specific expression of the transgene, and using Northern blot analysis with RNA isolated from the femura of the transgenic animals they found that the expression was at lest 20-fold increased compared to the expression in the bone of wildtype animals [6]. *Lrp5*-deficient mice (*Lrp5^−/−^*) mice (*C57BL/6* genetic background) were provided by Jackson Laboratory (005823, Bar Harbor, Maine, USA). Mice were kept in individual cages with a 12 h circadian rhythm and were given ad libitum access to food and water.

### Fracture healing study

Female, 26 weeks old mice (n = 78) of each genotype (n = 22–30, 25±3 g body weight) were used for the study. For investigation of fracture healing at day 21 the mice were randomly divided into three groups (*C57BL/6* wildtype, *Lrp5^−/−^* and *Col1a1-Krm2* mice) with either semi-rigid (n = 7–11/group) or flexible fracture fixation (n = 4–7/group) in order to generate mechanical conditions inducing regular or delayed healing, respectively [21]. Motion and ground reaction forces were monitored during the healing period in order to control proper limb loading [21]. 21 days after surgery the mice were euthanized by carbon dioxide inhalation. The fracture calli of each genotype with either semi-rigid (n = 7–11) or flexible fracture fixation (n = 4–7) were evaluated at day 21 by biomechanical and histological evaluation and by micro-computed tomography (µCT). Additional animals of each genotype with semi-rigidly fixated osteotomy were sacrificed 10 days after surgery for a genome-wide comparative gene expression analysis of the fracture callus (n = 5–6) and for histological and immunohistological (n = 5–6) evaluation.

### Surgical procedure

All animals received an analgesic (15 mg/kg, Tramal, Gruenenthal GmbH, Aachen, Germany) subcutaneously during the operation and in the drinking water (25 mg/l) for the first 3 postoperative days. After a subcutaneous injection of atropine sulfate (50 μg/kg, Atropin, Braun, Melsungen, Germany) the mice were anesthetized with 2% isoflurane (Forene, Abbott, Wiesbaden, Germany). For antibiosis the animals received daily subcutaneous injections of clindamycin-2-dihydrogenphosphate (45 mg/kg, Clindamycin, Ratiopharm, Germany) until the 3^rd^ postoperative day. Penetrating the fascia lata between the gluteus superficialis and biceps femoris muscles the femur was exposed. The fixator was fitted in a cranio-lateral position using 4 mini-Schanz screws (Research Implant System, RIS, Davos, Switzerland), placing the 1st and 2nd screw proximal and distal of the trochanter tertius, respectively. In order to provoke regular or delayed bone healing a semi-rigid (axial stiffness 18.1 N/mm) or flexible (axial stiffness 0.82 N/mm) fixator was used [21]. A standardized osteotomy gap of 0.5 mm was created at the mid-shaft of the right femur in the middle between the inner screws by using a 0.45 mm Gigli saw. The muscles were sutured with absorbable (Vicryl, J&J, Norderstedt, Germany), and the skin with nonabsorbable thread (Resolon, Resorba, Nuernberg, Germany). All mice were allowed to move freely immediately post-surgery. Mice were sacrificed 10 or 21 days postoperatively using carbon dioxide inhalation.

### Biomechanical testing

In order to determine the mechanical properties of the fracture callus the bending stiffness was analyzed by a non-destructive 3-point bending test after a healing period of 21 days as described before [21]. Briefly, the proximal end of the femur was fixed in an aluminum cylinder with SelfCem (Heraeus Kulzer, Hanau, Germany). The cylinder itself was fixed in a hinge joint, serving as the proximal support for the bending test. The femoral condyles rested on the bending support, the distance between both supports being 20 mm (l). The bending load F was applied on the midshaft and continuously recorded versus sample deflection (d) up to a maximum force of 5 N. Since the callus was not always located in the middle of the supports (l/2), the distances between the load vector and the proximal (a) and distal (b) supports were considered for calculating the bending stiffness EI = k((a^2^b^2^)/3 l) [21].

### µCT scanning

At day 21 post fracture the femora were imaged at a resolution of 30 µm, using a µCT Fan Beam Yscope System (Stratec Medizintechnik GmbH, Pforzheim, Germany) operating at a peak voltage of 40 kV and 140 µA. 3D reconstructions were visualized using 3D software (VG Studio Max 1.0; Volume Graphics, Heidelberg, Germany) and total volume (TV), maximum moment of inertia (I_max_), and bone volume fraction (BV/TV) were measured after segmentation of the former osteotomy gap. Global thresholding was performed to distinguish between mineralized and non-mineralized tissue. The gray value corresponding to 25% of x-ray attenuation of the cortical bone of each specimen was taken as threshold [21].

### Histology

The fracture calli harvested 21 days after surgery were processed for undecalcified histology. The bones were fixed in 4% formaldehyde, dehydrated by increasing ethanol concentrations and embedded in methyl methacrylate. 70 µm sections were cut and surface stained with Paragon (Paragon C&C, New York, NY). The newly formed callus was examined qualitatively and quantitatively under light microscopy (Axiophot; Zeiss, Oberkochen, Germany). The relative amounts of bone, cartilage, and soft tissue were determined at the osteotomy gap by the point counting method. Additionally, bony bridging of the fracture gap was evaluated in the histological slides at 4 positions at the fracture gap: anterior and posterior callus each peripherally (periosteal callus) and in between the cortices (intracortical callus). To describe the quality of bony bridging we used a scoring system (4 =  complete bony bridging at all 4 locations; 3 =  at 3 locations; 2 =  at 2 locations; 1 =  at 1 location; 0 =  no bony bridging).

Additional fracture calli were harvested 10 days after surgery from rigidly fixated groups and processed for decalcified histology. The specimens were fixed in 4% formaldehyde, decalcified in 20% EDTA and embedded in paraffin. Longitudinal sections of 5–7 µm were stained by Giemsa (Merck, Darmstadt, Germany) and the relative amounts of osseous tissue, cartilage, and fibrous tissue were determined as described above.

### Immunohistochemistry

Osteoclasts were identified by histochemical staining of tartrat-resistant acid phosphatase (TRAP) (Leucocyte Acid Phosphatase Kit, Sigma Aldrich Chemie GmbH, Steinheim, Germany) and counted in the whole peripheral callus. Additional paraffin sections were used for immunostaining of sphingomyelin phosphodiesterase 3 (Smpd3) by the use of a polyclonal anti-mouse Smpd3 antibody (Santa Cruz Biotechnology Inc., Heidelberg, Germany). To detect the primary antibody, labeled-streptavidin-biotin method (LAB-SA, Histostain-Plus Kit, Life Technologies GmbH, Darmstadt, Germany) was used and 3-amino-9-ethylcarbazole (AEC) was used as chromogen (Zytomed Systems, Berlin, Germany). The monoclonal non-phospho (active) β-catenin antibody (Cell Signaling, Danvers, MA, USA) and the polyclonal anti-collagen type II antibody (Rockland, Gilbertsville, PA, USA) were used together with the Vectastain ABC kit (Vector Laboratories, Burlingham, CA, USA) to detect the stabilized active form of endogenous β-catenin and collagen type II, respectively. Finally, counterstaining with hematoxylin (Waldeck, Münster, Germany) was performed. The sections were qualitatively evaluated under light microscopy.

### Whole transcriptome analysis of the fracture callus

Three mice of each wildtype, *Lrp5^−/−^* and *Col1a1-Krm2* with semi-rigidly fixated osteotomy were sacrificed 10 days after operation. Whole femur was dissected and immediately shock frozen in liquid nitrogen. Callus region (in the following referred to as callus) between 2^nd^ and 3^rd^ pin hole was homogenized using a knife-rotor homogenizator (T 10 basic ULTRA-TURRAX, IKA-Werke GmbH & Co. KG, Staufen, Germany). Total RNA was isolated with RNeasy Lipid Tissue Mini Kit (Qiagen, Hilden, Germany) according to manufacturer's instructions. Following amplification, labeling and fragmentation according to the Gene Chip 3′IVT Express Kit (Affymetrix, High Wycombe, United Kingdom), 10 µg of cRNA were hybridized on Affymetrix Gene Chips Mouse Genome 430 2.0. Hybridization signals were detected by Affymetrix Gene Chip Scanner 3000 and global normalization was performed by Affymetrix Gene Chip Operating Software 1.4 using the MAS5 algorithm. Normalized data of three calli of wildtype mice were crosswise compared to three calli of *Lrp5^−/−^* mice and *Col1a1-Krm2* mice, respectively. Statistical analysis was performed by Partek Genomic Suite (Partek Incorporated, St. Louis, USA) using ANOVA test with multiple testing corrections. The complete data were deposited in NCBI's Gene Expression Omnibus (GEO, http://www.ncbi.nlm.nih.gov/geo/) and are accessible through GEO SuperSeries accession number GSE51686. Differential gene expression in the comparisons *Lrp5^−/−^* versus wildtype and *Col1a1-Krm2* versus wildtype was regarded as significantly reliable when gene products (probe sets) fulfilled the following criteria: present calls in all three samples of at least one of the compared groups; change p-value <0.002 for the increased expression and p-value >0.998 for the decreased expression in at least 7 of the 9 cross-comparisons. Categorization of genes into functional groups was done by using Gene Ontology (GO) classification. (http://www.geneontology.org/).

### Quantitative real-time PCR

For cDNA synthesis we used 1 µg of total RNA of fracture calli harvested from wildtype mice (n = 6), *Lrp5^−/−^* mice (n = 6) and *Col1a1-Krm2* mice (n = 5) including the 3 RNA samples of microarray hybridization. Reverse transcription was performed with Oligo(dT)15 primers (Peqlab Biotechnologie GmbH, Erlangen, Germany) using MMLV reverse transcriptase (Promega GmbH, Mannheim, Germany) according to the manufacturer's instructions. Quantitative real-time PCR (qPCR) was performed using KAPA SYBR FAST Universal 2xqPCR Master Mix (Peqlab Biotechnologie GmbH) and 0.25pmol of sequence specific primers obtained from biomers.net GmbH (Ulm, Germany) (Table 1). Results were calculated with the efficiency-corrected Ct model [22] with B2m as the house-keeping gene. Groups were compared with non-parametrical Mann-Whitney U test.

**Table 1:** Quantitative PCR primer sequences.

Gene name	Forward primer (5′-3′)	Reverse primer (5′-3′)	NCBI Accession No.
Alpl	aacccagacacaagcattcc	gagagcgaagggtcagtcag	NM_007431.2
B2m	gtctttctggtgcttgtctc	agttcagtatgttcggcttc	NM_009735.3
Col1a1	ggcaagaatggagatgatgg	accatccaaaccactgaagc	NM_007742.3
Cyclin D1	ttgactgccgagaagttgtg	ctggcattttggagaggaag	NM_007631.2
Cyclin E1	acagcttggatttgctggac	actgtctttggaggcaatgg	NM_007633.2
Pth1r	accccgagtctaaagagaac	taaatgtaatcgggacaagg	NM_001083936.1
Tnfrsf11b	tgttccggaaacagagaagc	actctcggcattcactttgg	NM_008764.3

### Statistics

The statistical methods used for analysis of the microarrays and qPCR were already described in the corresponding paragraphs above. Results are presented as mean and standard deviation (SD). The number of animals included in the different groups (three genotypes with either flexible or more rigid fixation) is indicated in the legends. Biomechanical, μCT, and histochemical data of the groups were compared with non-parametrical Mann-Whitney U test using PASW Statistics 18.0 software (SPSS Inc., Chicago, USA). Level of significance was p≤0.05.

## Results

### Alterations in fracture healing do not result from differing limb loading

**Figure 1:**
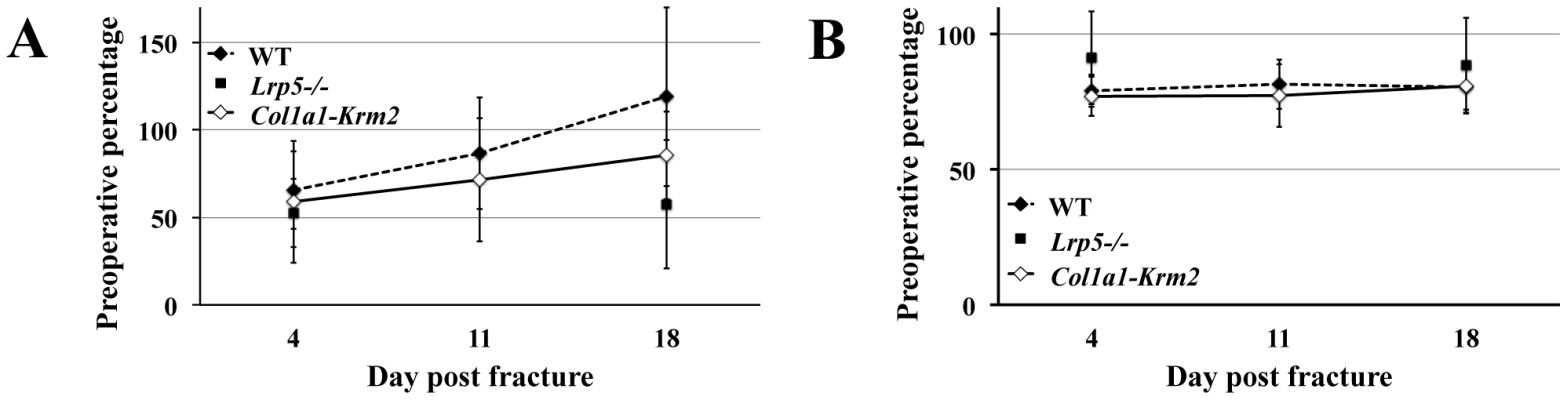
The different genotypes do not affect limb loading during fracture healing. (A) Motion of the mice was measured pre- and postoperatively using an infrared beam detection system. (B) Peak vertical ground reaction forces of the operated limb were recorded during movement of the mice using a force plate. n = 3–7 randomly selected mice per group (wildtype: dashed line (n = 7), *Lrp5^−/−^*: without line (n = 3), *Col1a1-Krm2*: black line (n = 6)). Postoperative values were related to preoperative measurements. Data are expressed as means ± SD.

In order to exclude that alterations in bone healing result from differing mechanical stimuli acting on the regenerating tissue we recorded the ground reaction forces of the operated limb and the activity of the *Lrp5^−/−^* and *Col1a1-Krm2* mice. The activity of the animals decreased postoperatively to about 70% of the preoperative value and then tended to increase to preoperative values with regard to wildtype mice (Fig. 1A). On average, the activities of *Lrp5^−/−^* and *Col1a1-Krm2* mice did not seem to reach these values. However, there were no significant differences between the three genotypes. The ground reaction forces of the operated limb postoperatively declined by about 20% and remained at this level during the entire healing period (Fig. 1B). We could not detect any significant differences between the genotypes. It can therefore be suggested that the osteoporotic mice loaded the operated limb properly, ensuring that alterations in bone healing are caused by the genotype and not by differing mechanical conditions in the fracture gap.

### *Krm2* overexpression in osteoblasts and *Lrp5* deficiency impair regular fracture healing

**Figure 2:**
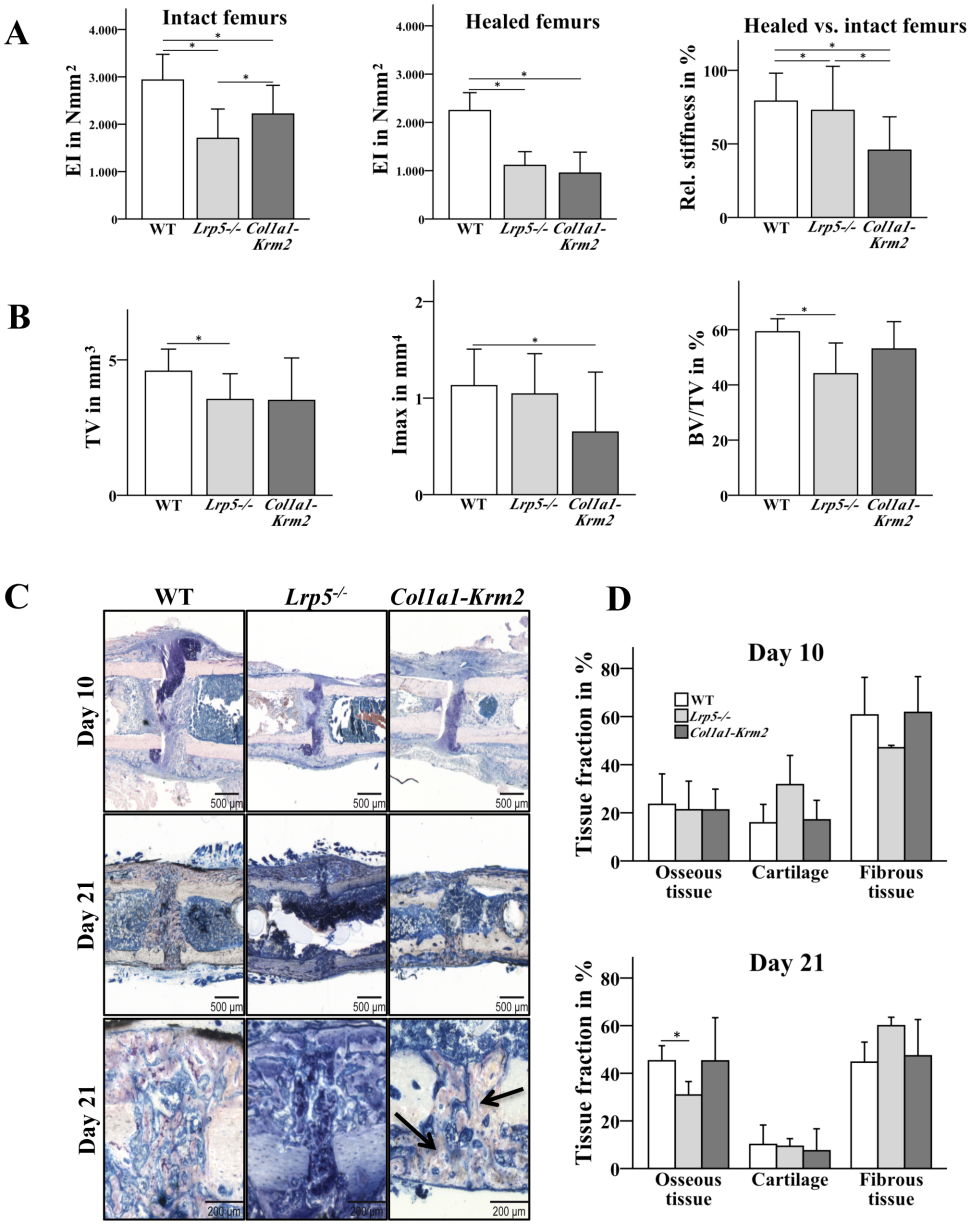
*Krm2* overexpression in osteoblasts and *Lrp5* deficiency impair regular fracture healing. The fractured femurs of wildtype, *Col1a1-Krm2* and *Lrp5^−/−^* mice were stabilized using a semi-rigid fixator in order to provide better mechanical healing conditions as described in the Material and Methods section. (A) In order determine the mechanical competence of the femurs the stiffness of both intact and fractured femurs was measured. Wildtype (n = 8), *Lrp5^−/−^* (n = 9), *Col1a1-Krm2* (n = 7) ± SD * p<0.05. (B) µCT analysis was performed to measure callus volume (TV), maximum moment of inertia (Imax) and bone volume fraction (BV/TV). Wildtype (n = 8), *Lrp5^−/−^* (n = 7), *Col1a1-Krm2* (n = 7) ± SD. * p<0.05 versus wildtype. (C) For histological analysis, the paraffin sections of the calli were stained with Giemsa at day 10 post fracture. Sections of methacrylate embedded calli were stained with Paragon at day 21 post fracture. The black arrows indicate crude and less branched bone trabeculae in the callus of *Col1a1-Krm2 mice* (D) Histomorphometric evaluation was performed to quantify total osseous tissue, cartilage, and fibrous tissue in the callus at day 10 and 21 post fracture. Wildtype (n = 8), *Lrp5^−/−^* (n = 5), *Col1a1-Krm2* (n = 5) ± SD. * p<0.05 versus wildtype.

To investigate fracture healing under better mechanical conditions we used a semi-rigid fixator, which has previously been shown to allow fast bone healing by promoting intramembranous with respect to endochondral bone formation [21]. As one important parameter for successful fracture healing we assessed the mechanical competence of both, the healed femurs at day 21 post fracture, the time point of bony bridging of the gap, as well as of the contralateral intact femurs. The stiffness of the intact *Lrp5^−/−^* and *Col1a1-Krm2* femurs was significantly decreased compared to the wildtype confirming their osteoporotic phenotype [6], [7] (Fig. 2A). The stiffness of the healed femurs was considerably reduced, by 50% in *Lrp5^−/−^*-deficient mice and by 58% in *Col1a1-Krm2* transgenic mice compared to wildtype controls (Fig. 2A). By comparing the stiffness of the osteotomized to the intact femurs we found that healed bones did not reach the mechanical competence of the intact bones in all mice strains at this time point of evaluation. The relative stiffness was most decreased in *Col1a1-Krm2* mice (Fig. 2A). These data were confirmed by the semiquantitative assessment of bony bridging of the osteotomy gaps. Whereas most of the wildtype mice exhibited complete bony bridging in the rigidly fixated groups, bridging was less successful in osteoporotic mice with the poorest results in *Col1a1-Krm2* mice (Table 2).

**Table 2:** Bony bridging of the fracture callus.

		Number of Mice with Score					
Fixation	Genotypes	0	1	2	3	4	Mean Score
Rigid	WT				2	5	3.7
	Lrp5*^−^*/*^−^*			3	2		2.5
	Col1a1-Krm2		1	4			1.8
Flexible	WT		2	2			1.5
	Lrp5*^−^*/*^−^*		1	3			0.7
	Col1a1-Krm2		1	3			0.7

Bony bridging of the fracture callus evaluated at 4 locations in the fracture callus: anterior and posterior callus peripherally (periosteal callus) and in between the cortices (intracortical callus). The quality of bone bridging was described by a scoring system (4 =  complete bony bridging at all locations; 3 =  at 3 locations; at 2 locations; 1 =  at 1 location; 0 =  no bony bridging). The table displays the number of mice with a distinct bridging score within the groups. n = 4–7 mice per group.

µCT measurements of the fracture calli revealed that the geometrical parameters (total callus volume, maximum moment of inertia) were slightly decreased in the rigidly fixated osteoporotic mouse strains compared to wildtype controls indicating inferior callus formation (Fig. 2B). The relative amount of mineralized tissue in the osteotomy gap (BV/TV) was significantly decreased in *Lrp5^−/−^* mice but not in *Col1a1-Krm2* mice indicating that the *Col1a1-Krm2* transgenic callus was small but transformed to mineralized tissue (Fig. 2B).

Histologically, we found a lot of cartilage near the osteotomy gap after 10 days, indicating secondary bone formation at this location. Intramembranous bone formation started near the periosteum in some distance to the gap (Fig. 2C). The pattern of tissue distribution was similar in all groups with the more rigid fixation. The histomorphometric data revealed no differences in the relative amount of bone and cartilage after 10 days (Fig. 2D). After 21 days, cartilage and fibrous tissue were decreased in favor of newly formed bone in all mice. Confirming the µCT data, the bone fraction was reduced in *Lrp5^−/−^* but not in *Col1a1-Krm2* mice. The newly formed bone trabeculae in the callus of the *Col1a1-Krm2* transgenic mice appeared crude and less branched and the osteoblasts covering the bone surfaces displayed an irregular morphology. These morphological abnormalities were not observed in *Lrp5^−/−^* mice (Fig. 2C).

### Fracture healing is more strongly impaired in *Col1a1-Krm2* mice than in *Lrp5^−/−^* mice

**Figure 3:**
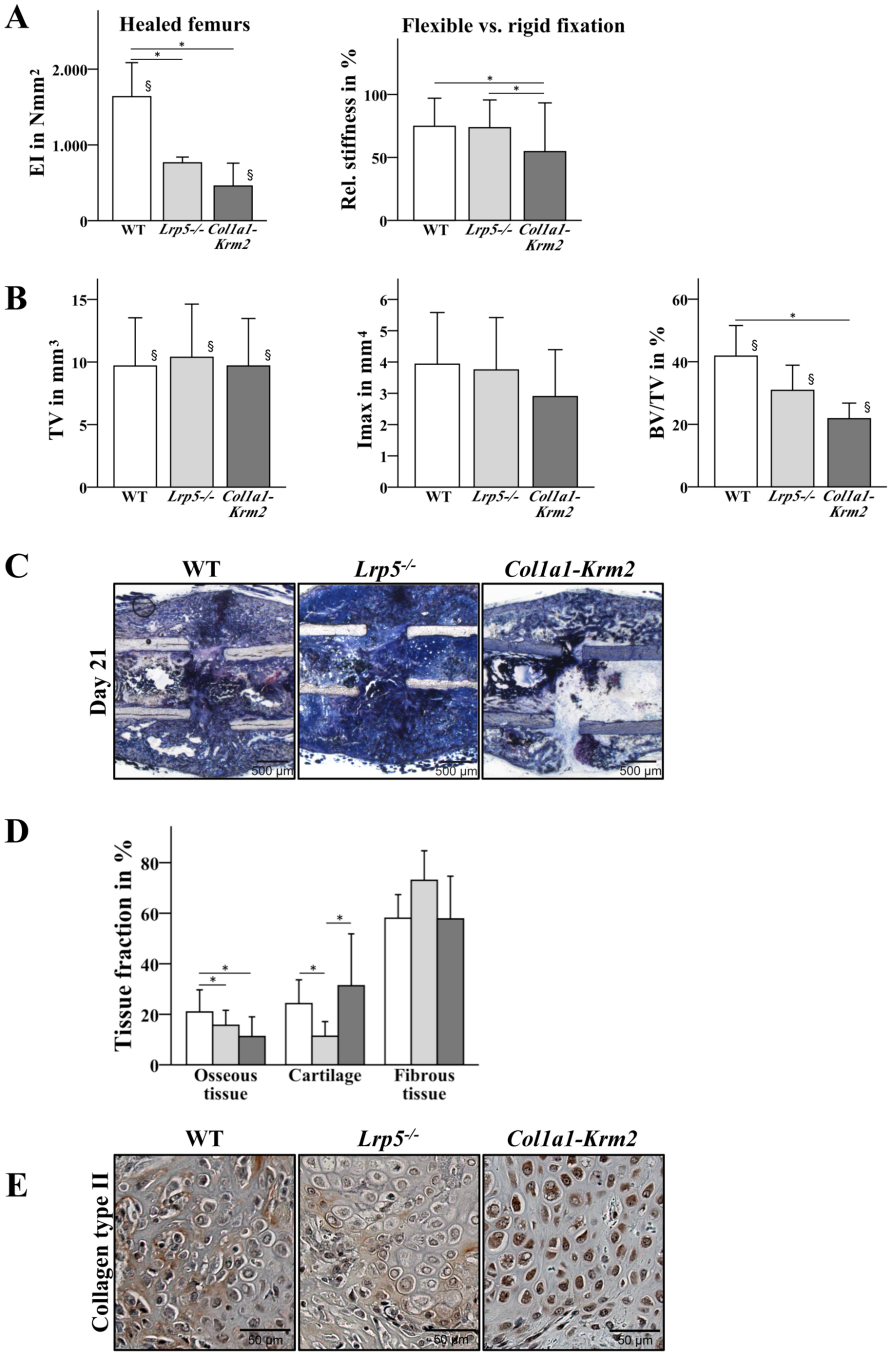
*Krm2* overexpression in osteoblasts lead to a more strongly impaired healing than Lrp5 deficiency. The fractured femurs of wildtype, *Col1a1-Krm2* and *Lrp5^−/−^* mice were stabilized using a flexible fixator in order to induce delayed healing as described in the Material and Methods section. (A) In order determine the mechanical competence of the femurs the stiffness of the fractured femurs was measured. (B) µCT analysis was performed to measure callus volume (TV), maximum moment of inertia (Imax) and bone volume fraction (BV/TV). Wildtype (n = 5), *Lrp5^−/−^* (n = 4), *Col1a1-Krm2* (n = 7) ± SD. * p<0.05 versus wildtype. § p<0.05 versus rigidly fixated group. (C) For histological analysis, sections of methacrylate embedded calli were stained with Paragon at day 21 post fracture. (D) Histomorphometric evaluation was performed to quantify total osseous tissue, cartilage, and fibrous tissue in the calli at day 21 post fracture. Wildtype (n = 5), *Lrp5^−/−^* (n = 4), *Col1a1-Krm2* (n = 5) ± SD. * p<0.05 versus wildtype. (E) Representative images of collagen type II expression detected by immunostaining at day 10 post fracture.

In the used mouse model, flexible fracture fixation provoked the formation of a large callus with inferior mechanical competence indicating delayed bone healing with predominantly endochondral bone formation [21]. The mechanically induced delay of fracture healing was also observed in *Lrp5^−/−^* and *Col1a1-Krm2* mice. The flexibilization of the fracture fixation reduced the callus stiffness by about 25% in wildtype as well as in *Lrp5^−/−^* mice with no significant differences between both genotypes, and by 46% in *Col1a1-Krm2* mice (Fig. 3A). The bony bridging of the callus was poor, particularly in the osteoporotic mice (Table 2). µCT analysis revealed that total callus volume and maximum moment of inertia were more than doubled in all flexibly fixated mice compared to the rigidly fixated groups with no significant differences between the genotypes (Fig. 3A). However, the mineralized fraction (BV/TV) of the calli of all genotypes was significantly decreased compared to the rigidly fixated groups indicating inferior bone formation under flexible conditions. Being in line with the mechanical data the reduction of the mineralized tissue fraction was most pronounced in the *Col1a1-Krm2* transgenic mice (Fig. 3B). The histomorphometrical data confirmed the µCT data (Fig. 3C, D). The cartilage fraction in the *Lrp5^−/−^* calli was reduced compared to *Col1a1-Krm2* and wildtype mice indicating reduced endochondral bone formation. This suggestion was supported by a lower expression of collagen type II in the calli of *Lrp5^−/−^* mice compared to *Col1a1-Krm2* and wild-type mice (Fig. 3E). Taken together the results revealed that bone healing was most compromised in *Col1a1-Krm2* mice under mechanically unstable conditions.

### Identification of differentially expressed genes in fracture calli of *Lrp5^−/−^* and *Col1a1-Krm2* mice

**Figure 4:**
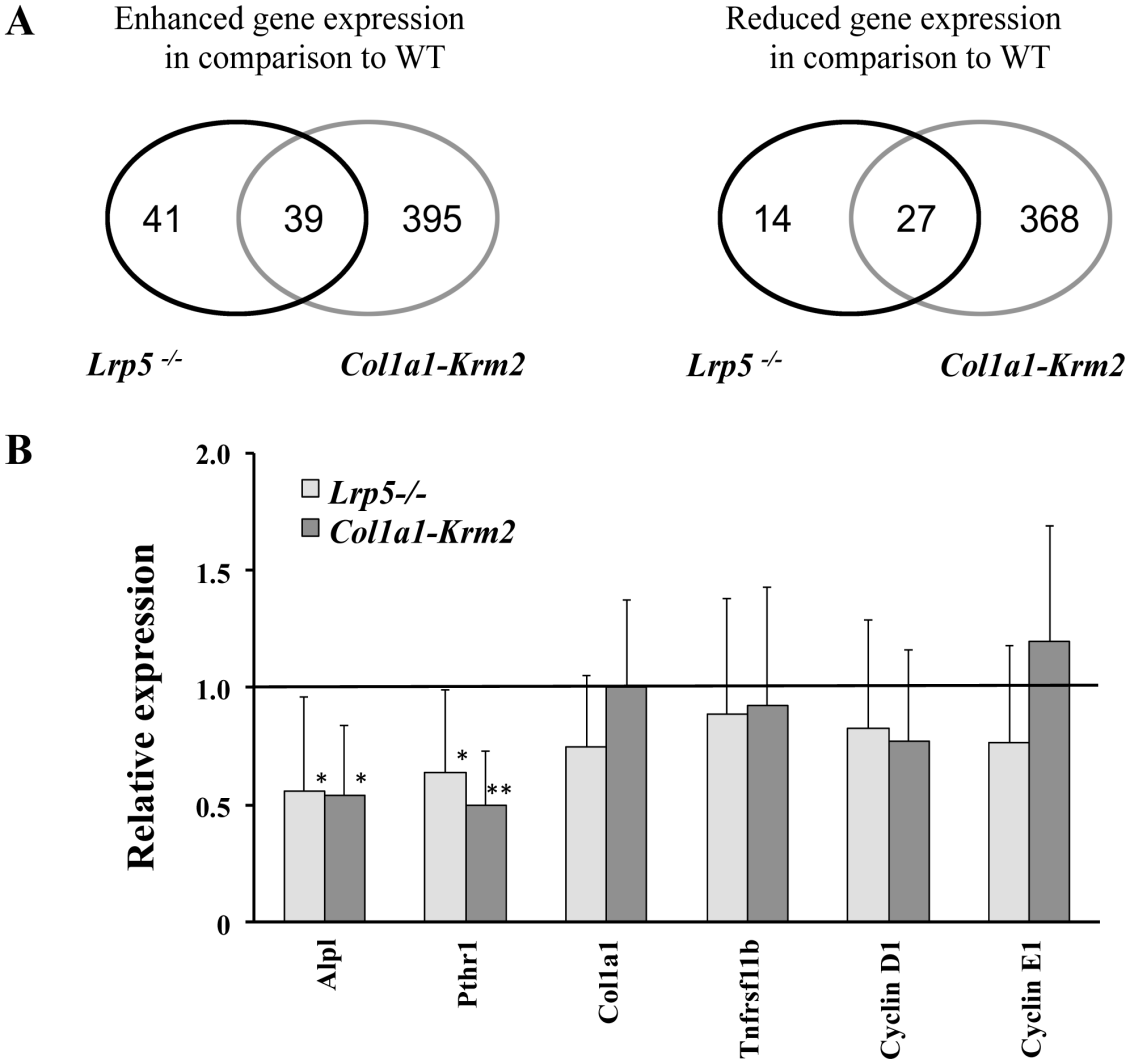
*Krm2* overexpression in osteoblasts has a stronger effect on gene expression than *Lrp5* deficiency. (A) The numbers indicate the number of gene products with significantly differential expression in calli of *Col1a1-Krm2* and *Lrp5-/-* mice in comparison to WT mice (for gene names see Table S1). (B) Relative changes in gene expression of osteoblast associated genes and Wnt target genes. QPCR was performed with samples prepared from callus tissue under semi-rigid fixation 10 days post-fracture. Gene expression in *Col1a1-Krm2* mice and *Lrp5^−/−^* mice are expressed as fold change relative to wildtype mice. Wildtype (n = 6), *Lrp5^−/−^* (n = 6), *Col1a1-Krm2* (n = 5) ± SD. * p<0.05, ** p<0.01.

To identify genes that may be responsible for the observed greater impairment of healing in *Col1a1-Krm2* mice compared to that in *Lrp5^−/−^* mice, we performed microarray analysis of three independent callus samples from each mouse strain in the semi-rigidly fixated group. We collected the samples 10 days after surgery, because endochondral and intramembraneous bone formation took place and β-catenin-mediated transcription has been shown to be active at this time point [13]. Gene expression profiles of *Lrp5^−/−^* and *Col1a1-Krm2* mice were each compared to wildtype mice. We obtained 121 differentially expressed gene products in calli of *Lrp5^−/−^* mice and 829 differentially expressed gene products in *Col1a1-Krm2* mice (Fig. 4A, Table S1). Some of the gene expression changes were identical in both datasets and the overlap comprised 39 gene products with increased expression and 27 gene products with reduced expression (including osteoblast associated genes *Alpl* and *Pthr1*) in both, *Col1a1-Krm2* and *Lrp5^−/−^* calli. We focused on differentially expressed genes associated with bone/ossification and cartilage, to identify possible candidate genes for the decreased fracture healing potential we observed (Table 3).

**Table 3:** Candidate genes for impaired fracture healing in *Col1a1-Krm2* and *Lrp5^−/−^* mice.

		Microarray				
			*Col1a1-Krm2* (n = 3)		*Lrp5^−/−^* (n = 3)	
Gene	Gene Title	Probe set ID	FC	SD	FC	SD
**Bone/Ossification**						
Alpl	alkaline phosphatase, liver/bone/kidney	1423611_at	0.57	0.24	0.66	0.23
Cd276	CD276 antigen	1417599_at	0.58	0.19	―	―
Col13a1	collagen, type XIII, alpha 1	1422866_at	0.61	0.28	―	―
Col1a2	collagen, type I, alpha 2	1446326_at	0.66	0.27	―	―
Ctse	cathepsin E	1418989_at	4.22	2.38	―	―
Egfr	epidermal growth factor receptor	1435888_at	0.62	0.17	―	―
Fgfr2	fibroblast growth factor receptor 2	1433489_s_at	0.61	0.26	―	―
Lrrc17	leucine rich repeat containing 17	1429679_at	0.62	0.15	―	―
Mmp14	matrix metallopeptidase 14	1416572_at	0.54	0.21	―	―
Mmp2	matrix metallopeptidase 2	1439364_a_at	0.63	0.25	―	―
Pbx1	pre B-cell leukemia transcription factor 1	1440037_at	1.91	0.59	―	―
Phex	phosphate regulating gene with homologies to endopeptidases on the X chromosome	1421979_at	0.71	0.24	―	―
Pth1r	parathyroid hormone 1 receptor	1417092_at	0.60	0.39	0.66	0.33
Smpd3	sphingomyelin phosphodiesterase 3, neutral	1422779_at	0.52	0.18	―	―
Tgfb2	transforming growth factor, beta 2	1450923_at	0.56	0.26	―	―
**Cartilage**						
Aspn	asporin	1416652_at	0.55	0.16	―	―
Col11a1	collagen, type XI, alpha 1	1449154_at	―	―	0.77	0.23

Differential gene expression in callus tissue was analyzed 10 days post-fracture by microarray analysis (n = 3). Only gene products that fulfilled criteria for significant gene expression changes (see methods) are listed.

FC: mean value of fold changes of the cross-wise comparisons with wildtype callus (n = 3) as determined by microarray analysis.

SD: standard deviation.

― no significantly differential expression when compared to wildtype callus (n = 3) as determined by microarray analysis.

We observed a markedly decreased expression of genes involved in chondrocyte and/or osteoblast differentiation as well as endochondral and intramembranous ossification, including *Egfr* [23], *Fgfr2* [24], *Mmp2* [25], *Mmp14* [26] and *Cd276* [27] in calli of *Col1a1-Krm2* mice, but not in Lrp5*^−^* ^/*−*^ mice. According to the results of the microarray hybridization, osteoblast marker *Col1a2* was significantly decreased in calli of *Krm2* over-expressing mice compared to wildtype. Another marker for osteoblastogenesis, *Alpl* was decreased in calli of *Col1a1-Krm2* transgenic and *Lrp5^−/−^* mice, whereas the microarray results did not reveal any reliable differences of other well-established osteoblast differentiation markers, such as *Runx2*, *Col1a1*, *Bglap* or *Spp1*.

We did not observe any alterations in the expression of *Tnfrsf11b*, which encodes for the RANKL antagonist osteoprotegerin that functions as an osteoclastogenesis inhibitor. The gene has been demonstrated to be regulated by canonical Wnt signaling [28] and was shown to be significantly reduced in primary osteoblasts of *Col1a1-Krm2* transgenic mice [6].

**Figure 5:**
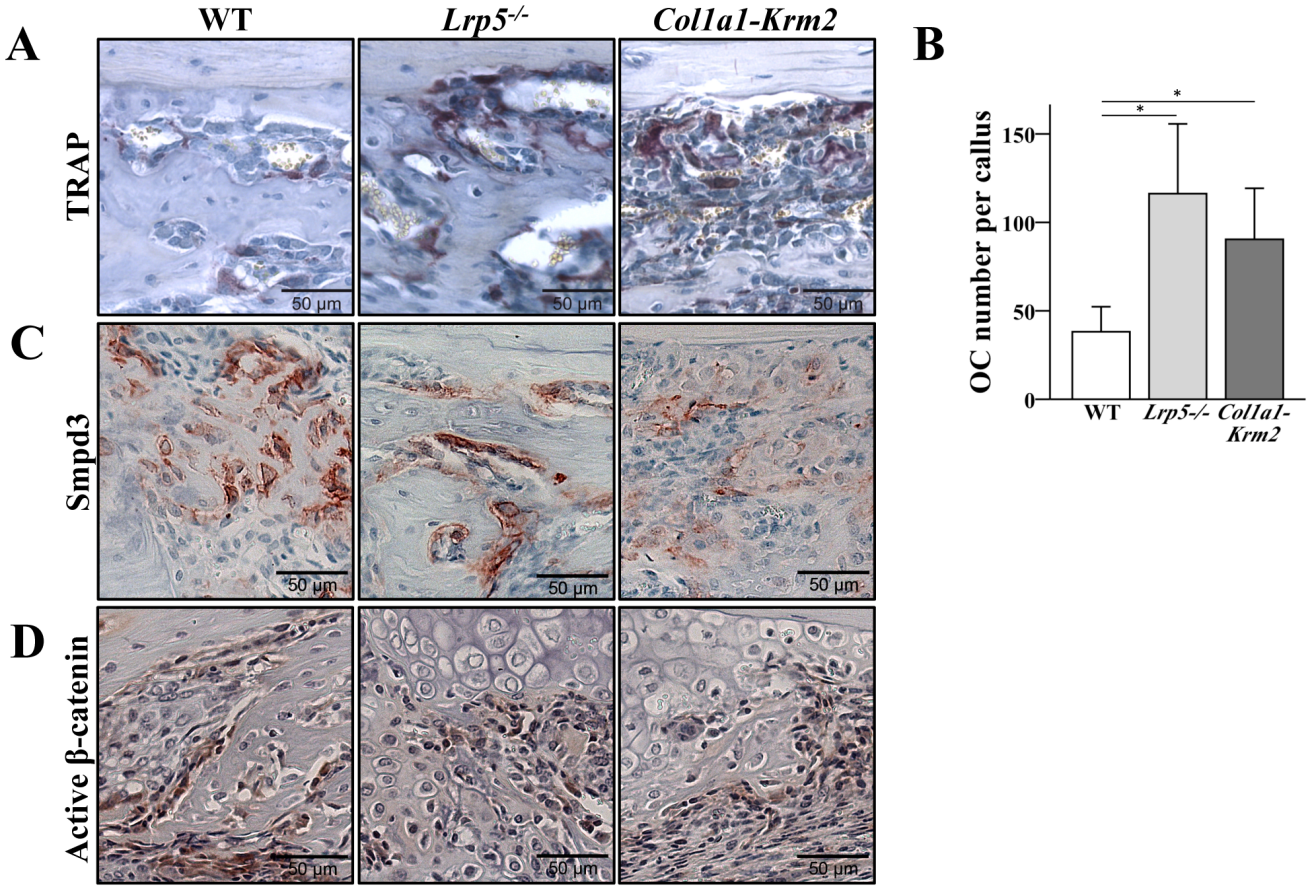
*Krm2* overexpression and *Lrp5* deficiency result in increased osteoclast number. (A) Osteoclasts were identified by histochemical staining of tartrat-resistant acid phosphatase (TRAP) at day 10 post fracture. (B) Osteoclast number was determined in the peripheral callus at day 10 post fracture. Wildtype (n = 6), *Lrp5^−/−^* (n = 4), *Col1a1-Krm2* (n = 5) ± SD. * p<0.05 versus wildtype. Representative images of (C) Smpd3 expression and (D) active β-catenin levels detected by immunostaining at day 10 post fracture.

Furthermore, our microarray data of *Col1a1-Krm2* calli indicated a significant decrease of *Phex* [29] and *Smpd3* [30] (Table 3), genes described as down-regulated in primary osteoblasts of this mouse strain [6]. The decreased expression of *Smpd3* is in line with the reduced immunohistological staining of Smpd3 protein that we found in *Col1a1-Krm2* transgenic calli (Fig. 5C). In wildtype mice, Smpd3 was mainly present in osteoblasts in the peripheral callus and to a less extend in chondroblasts and precursor cells in the immature tissue in the osteotomy gap. Staining intensity and pattern was similar in *Lrp5^−/−^* calli. The sphingomyelinase Smpd3 and the product of its enzymatic action on sphingomyelin, ceramide, are inhibitory factors of bone resorption [31].

Increased expression of *Ctse* that encode for cathepsin E, respectively, indicates enhanced osteoclast activity or number in *Col1a1-Krm2* mice in comparison with wildtype mice [32]. Since it has been shown that canonical Wnt signaling affects bone resorption, we analyzed osteoclast number in the calli of the three different genotypes. We found a significantly enhanced number of TRAP-positive osteoclasts in the peripheral callus of *Lrp5^−/−^* as well as *Col1a1-Krm2* mice (Fig. 5A, B).

Only few genes linked to cartilage were differentially expressed in comparison with wildtype, including Col11a1 that was decreased in *Lrp5^−/−^* mice (Table 3). Decrease of *Col11a1* indicates impaired cartilage collagen fibril formation and extracellular matrix formation [33], which may reflect impaired enchondral bone formation.

It was reported that the only genes whose expression was decreased in bone of *Lrp5^−/−^* mice were the regulators of cell proliferation *CylcinD1*, *D2* and *E1* [34]. We analyzed two of those genes by qPCR and did not detect differential expression in calli of *Lrp5^−/−^* or *Col1a1-Krm2* (Fig. 4B), which might be due to the different origin of the RNA used in our approach.

As β-catenin has been shown to be a key downstream mediator in canonical Wnt/β-catenin signaling (26), we analyzed the occurrence of active β-catenin in the calli of the different genotypes. Unphosphorylated stabilized β-catenin was predominantly present in osteoblastic cells lining the periosteum and newly formed trabeculae as well as in prehypertrophic chondrocytes. Staining intensity was lowest in *Col1a1-Krm2* calli (Fig. 5D). However, *Lrp5^−/−^* calli showed also decreased levels of active β-catenin in comparison with its occurrence in the calli of wild-type mice.

## Discussion

In the present study, we investigated the influence of *Lrp5* deficiency and osteoblast-specific overexpression of *Krm2* in fracture healing using *Lrp5^−/−^* and *Col1a1-Krm2* mice. Our data indicated that fracture healing was highly impaired in both mouse genotypes, but that osteoblast-specific *Krm2* overexpression resulted in an even worse healing phenotype than *Lrp5* deficiency. Moreover, our data revealed that the stronger impairment of healing in *Col1a1-Krm2* was associated with decreased expression of genes mainly involved in osteogenesis.

Although various findings of mouse genetic studies demonstrated the crucial influence of Wnt signaling on bone mass maintenance, the precise biological role of the regulatory molecules in this process has not yet been fully elucidated. Thus, in particular the biological role of *Lrp5* in bone remodeling is controversially discussed [34], [35]. Although a positive role has been reported for *Krm2* on *Lrp6*-mediated Wnt signaling, which is presumably facilitated through an interaction with the Wnt signaling agonists of the R-Spondin (Rspo) family [36], the antagonistic effect of Krm2 by binding to the Dkk1 receptors on canonical Wnt signaling has been well described already before [9].

In the present study on bone regeneration in *Lrp5^−/−^* and *Col1a1-Krm2* mice, biomechanical testing of the fracture calli of the semi-rigidly fixated groups and comparison of the stiffness of the fractured femur with the stiffness of the respective genotype-specific intact femur revealed a more impaired healing in *Col1a1-Krm2* mice than in *Lrp5^−/−^* mice. This fact was supported by analysis of bony bridging of the osteotomy gaps, which was poorest in the *Col1a1-Krm2* group. µCT analysis (bone tissue fraction, maximum of inertia, tissue volume) of the semi-rigidly fixated groups revealed that the calli of the *Col1a1-Krm2* and *Lrp5^−/−^* mice comprised a similarly decreased amount of mineralized tissue fraction, but *Col1a1-Krm2* calli seemed to be smaller compared to the calli of wild-type mice. Moreover, histological assessment of the fracture calli showed a stronger impairment of bone regeneration in *Col1a1-Krm2* transgenic mice, as the bone trabeculae appeared crude and less branched and the osteoblasts covering the bone surfaces displayed an irregular morphology.

We have previously shown that both external fixators, which were used in the present study, allowed fracture healing under controlled rigid and flexible conditions, and that the more flexible fixation resulted in delayed bone healing compared to the more rigid fixation [21]. In the present study the differences in healing between the two genotypes became particularly evident in mice in which the more flexible fixation was used. The more flexible fixation revealed a significantly reduced bone tissue fraction in *Col1a1-Krm2* mice compared to wild-type mice. These results are consistent with our histological findings regarding the amount of bone that was significantly more reduced in *Col1a1-Krm2* mice than in *Lrp5^−/−^* mice compared to wild-type mice, when using the more flexible fixation and consistent with the observation that *Col1a1-Krm2* mice exhibited a significantly more severe osteoporotic phenotype than *Lrp5^−/−^* mice that has been shown to be due to increased bone resorption as well as disturbed osteoblast maturation and bone formation [6], [7]. Our data from the flexible group at day 21 indicated that cartilage tissue was significantly more increased in *Col1a1-Krm2* than in *Lrp5^−/−^* mice. We assume that in *Col1a1-Krm2* mice less bone tissue might result, at least in part, from increased cartilage production and mainly from reduced intramembranous bone formation. The differentiation of osteoblast precursor cells into the osteogenic lineage should be impaired by osteoblast-specific *Krm2* overexpression and instead these cells could convert into chondrogenic cells. These assumptions are in line with previous studies demonstrating that in osteoblast precursors lacking β-catenin osteogenic differentiation is inhibited and these cells develop into chondrocytes [17].

*Ex vivo* studies on *Col1a1-Krm2* osteoblasts suggested a cell-autonomous differentiation defect underlying the osteoporotic phenotype of *Col1a1-Krm2* mice and indicated a reduced Wnt3a-dependent activation of canonical Wnt signaling among other by decreased total β-catenin levels in *Col1a1-Krm2* osteoblasts compared to wild-type osteoblasts.

The latter finding is in line with the results of the present study because immunohistological validation of active β-catenin levels in the callus tissue revealed less active β-catenin in the calli of *Col1a1-Krm2* mice than in calli of wild-type mice. Active β-catenin was predominantly detected in cells surrounding the cartilage, in prehypertrophic chondrocytes as well as in osteoblasts lining the periosteum and the woven bone. Hypertrophic chondrocytes and osteoblasts, more deeply embedded in the bone matrix did not reveal significantly active β-catenin staining. The callus tissue of *Lrp5^−/−^* mice revealed a similar staining distribution to that in wild-type mice. However, in *Lrp5^−/−^* mice, overall active β-catenin staining was lower than in wild-type mice.

These findings are consistent with the results from a former study on bone regeneration in mice which provided evidence of active canonical Wnt signaling in intramembranous and endochondral ossification [37] and possibly account for the decreased intramembranous bone formation in *Col1a1-Krm2* and *Lrp5^−/−^* mice as well as the significantly diminished endochondral bone formation that was seen especially in *Lrp5^−/−^* mice with the more flexible fixation in the present study. Cartilage tissue was not significantly altered in *Lrp5^−/−^* mice treated with the more rigid fixation at day 10 of healing compared to *Col1a1-Krm2* and wildtype mice. However, in *Lrp5^−/−^* mice with the flexible fixation cartilage tissue at day 21 was signficantly decreased compared to *Col1a1-Krm2* as well as to wildtype mice. This result suggests that *Lrp5* deficiency did not result in increased or prolonged cartilage production compared to *Col1a1-Krm2* and wildtype mice. Reduced bone tissue in these mice seemed to be due to decreased endochondral as well as intramembranous bone formation. This is in line with previous studies demonstrating that mice conditionally expressing β-catenin null alleles had a lack of bone as well as cartilage and showed immature mesenchymal cells persisting at the fracture sites [13]. Mesenchymal cells that begin to show phenotypic features of either chondrocyte or osteoblast precursors exhibit β-catenin mediated transcription [13]. Thus, *Lrp5^−/−^* mice that are characterized by mesenchymal stem cells with less β-catenin signaling due to Lrp5 deficiency showed less chondogenic as well as osteogenic tissue with the flexible fixation in the present study. We detected less staining of collagen type II in prechondrogenic mesenchymal stem cells and chondogenic cells in the calli of *Lrp5^−/−^* mice compared to *Col1a1-Krm2* and wildtype mice. The latter finding together with the altered staining for active β-catenin detected in the calli of *Lrp5^−/−^* mice suggests an important role for *Lrp5* as Wnt-coreceptor in endochondral bone formation.

It has been shown that Kremen proteins decrease Wnt/β-catenin signaling by binding to Dkk1 [9] and that Tnfrsf11b, the gene encoding the osteoclast-inhibitory factor Opg, being known to be induced by canonical Wnt signaling in osteoblasts [28], is downregulated in *Col1a1-Krm2* mice [6], explaining the increased bone resorption and osteolytic lesions in the lower extremities in these mice. In contrast, bone resorption seemed to be unaffected in *Lrp5^−/−^* mice [7] and studies on mice expressing a mutant Lrp5 gene associated with high bone mass (HBM) demonstrated no significant changes in osteoclast number and function [4], suggesting that Lrp5 is not directly involved in regulating osteoclastogenesis and osteoclast activity. However, a more recent study showed an increase of the osteoclast surface in mice with a lack in Lrp5, implying that bone resorption might be affected by Lrp5 deficiency [38]. This would be consistent with our findings, which did not only reveal the expected significantly increased osteoclast number in the calli of *Col1a1-Krm2* mice, but also in the calli of *Lrp5^−/−^* mice, as assessed by histochemical staining of TRAP positive cells. In line with the results of Schulze et al. [6] regarding the expression of the sphingomyelinase *Smpd3*, that has been identified as an inhibitory factor of bone resorption [31] and has been shown to be downregulated in *Col1a1-Krm2* osteoblasts [6], we detected less expression of *Smpd3* in the calli of *Col1a1-Krm2* mice as verified by immunohistochemistry and microarray expression analysis. However, *Smpd3* expression did not seem to be decreased in the calli of *Lrp5^−/−^* mice. Nevertheless, the altered staining for active β-catenin in the fracture calli of *Lrp5^−/−^* mice suggests that *Lrp5* acts on canonical Wnt signaling in osteoblasts, which is compatible with the finding of a systemic influence of Lrp5 on bone formation. Interestingly, patients suffering from a non-classical form of osteopetrosis, the autosomal dominant osteopetrosis type I (ADOI), which is caused by a specific gain-of-function mutation in the LRP5 gene and is associated with a high bone mass phenotype, have abnormally low number of osteoclasts *in vivo* [39]. Results from later studies on osteoclasts from theses patients strongly suggested that the specific ADOI phenotype is caused by the reduced ability of osteoblasts to support osteoclast development due to increased β-catenin-dependent *Opg* expression [40], implying that *Lrp5* might regulate canonical Wnt signaling and indirectly osteoclast differentiation by modulation *Opg* expression.

Skeletal defects in mice caused by *Lrp5* loss-of-function mutations manifest primarily during the postnatal period [7], whereas *Lrp6*-deficient mice are not viable, underlining the particular role of *Lrp6* in embryogenesis [41]. However, it has already been shown that *Lrp5* and *Lrp6* exert overlapping functions in the control of postnatal bone mass accrual [42]–[44], and since *Lrp5* and *Lrp6* are highly homologous and are coexpressed in primary osteoblasts it can be assumed that *Lrp6* is able to compensate for *Lrp5* function during some stages of osteoblast differentiation [35]. This compensation might be the reason for the less impaired healing that was observed in *Lrp5^−/−^* mice compared to *Col1a1-Krm2* mice in our study. Since *Krm2* is acting by binding to *Dkk1* that can inhibit Wnt signaling through a direct interaction with both, *Lrp5* and *Lrp6*, a poorer healing in *Col1a1-Krm2* mice might be expected due to a more pronounced decrease of bone formation and increased bone resorption in these mice. To address this hypothesis we performed microarray analysis, using RNA from calli of each mouse genotype to identify genes that might be responsible for the observed differences in healing in the two genotypes. In the calli of both mouse genotypes we observed a significantly decreased expression of genes associated with osteogenesis, including *Alpl* and *Col1α2*, however, more ossification-related genes showed decreased expression in the calli of *Col1a1-Krm2* mice. Moreover, expression of the gene, encoding the gastric aspartyl protease cathepsin E (*Ctse*) that has also been found to be expressed in active osteoclasts [32], was significantly upregulated only in the calli of *Col1a1-Krm2* mice, an observation that is in line with our finding of enhanced osteoclast number in *Col1a1-Krm2* mice. The latter finding and the reduced expression of more osteogenesis-associated genes might at least be in part responsible for the more impaired bone healing observed in *Col1a1-Krm2* mice compared to *Lrp5^−/−^* mice.

In conclusion, our data underscore the important role of canonical Wnt signaling in bone formation as well as in drug targeting approaches, both in low bone mass diseases and in impaired fracture healing. Moreover, our data confirmed that activation of canonical β-catenin signaling as a therapy for fracture healing improvement should be restricted to mesenchymal precursor cells already committed to the osteogenic lineage. Since *Krm2* plays a crucial role in regulating bone formation, antagonizing KRM2 during a defined time of fracture healing might be an interesting option to improve fracture healing under compromised conditions, such as osteoporosis.

## Supporting Information

Table S1 **Differentially expressed gene products in fracture calli of** ***Col1a1-Krm2*** **and** ***Lrp5^−/−^*** **mice.** Differential gene expression in callus tissue under semi-rigid fixation was analyzed 10 days post-fracture by microarray analysis (n  =  3 each). Only gene products that fulfilled criteria for significant gene expression changes (see methods) are listed. FC: mean value of fold changes of the cross-wise comparisons with wildtype callus. SD: standard deviation. ― no significantly differential expression when compared to wildtype callus.
(PDF)

## References

[1] Baron R, Kneissel M (2013) WNT signaling in bone homeostasis and disease: from human mutations to treatments. Nat Med 19: 179–192. 23389618 10.1038/nm.3074

[2] Gong Y, Slee RB, Fukai N, Rawadi G, Roman-Roman S, et al (2001) LDL receptor-related protein 5 (LRP5) affects bone accrual and eye development. Cell 107: 513–523. 11719191 10.1016/s0092-8674(01)00571-2

[3] Zhang Y, Wang Y, Li X, Zhang J, Mao J, et al (2004) The LRP5 high-bone-mass G171V mutation disrupts LRP5 interaction with Mesd. Mol Cell Biol 24: 4677–4684. 15143163 10.1128/MCB.24.11.4677-4684.2004 PMC416395

[4] Babij P, Zhao W, Small C, Kharode Y, Yaworsky PJ, et al (2003) High bone mass in mice expressing a mutant LRP5 gene. J Bone Miner Res 18: 960–974. 12817748 10.1359/jbmr.2003.18.6.960

[5] Hoeppner LH, Secreto FJ, Westendorf JJ (2009) Wnt signaling as a therapeutic target for bone diseases. Expert Opin Ther Targets 13: 485–496. 19335070 10.1517/14728220902841961 PMC3023986

[6] Schulze J, Seitz S, Saito H, Schneebauer M, Marshall RP, et al (2010) Negative regulation of bone formation by the transmembrane Wnt antagonist Kremen-2. PLoS One 5: e10309. 20436912 10.1371/journal.pone.0010309 PMC2860505

[7] Kato M, Patel MS, Levasseur R, Lobov I, Chang BH, et al (2002) Cbfa1-independent decrease in osteoblast proliferation, osteopenia, and persistent embryonic eye vascularization in mice deficient in Lrp5, a Wnt coreceptor. J Cell Biol 157: 303–314. 11956231 10.1083/jcb.200201089 PMC2199263

[8] van der Horst G, van der Werf SM, Farih-Sips H, van Bezooijen RL, Lowik CW, et al (2005) Downregulation of Wnt signaling by increased expression of Dickkopf-1 and -2 is a prerequisite for late-stage osteoblast differentiation of KS483 cells. J Bone Miner Res 20: 1867–1877. 16160745 10.1359/JBMR.050614

[9] Mao B, Wu W, Davidson G, Marhold J, Li M, et al (2002) Kremen proteins are Dickkopf receptors that regulate Wnt/beta-catenin signalling. Nature 417: 664–667. 12050670 10.1038/nature756

[10] Ellwanger K, Saito H, Clement-Lacroix P, Maltry N, Niedermeyer J, et al (2008) Targeted disruption of the Wnt regulator Kremen induces limb defects and high bone density. Mol Cell Biol 28: 4875–4882. 18505822 10.1128/MCB.00222-08 PMC2493355

[11] Whyte JL, Smith AA, Helms JA (2012) Wnt signaling and injury repair. Cold Spring Harb Perspect Biol 4: a008078. 22723493 10.1101/cshperspect.a008078 PMC3405869

[12] Chen Y, Alman BA (2009) Wnt pathway, an essential role in bone regeneration. J Cell Biochem 106: 353–362. 19127541 10.1002/jcb.22020

[13] Chen Y, Whetstone HC, Lin AC, Nadesan P, Wei Q, et al (2007) Beta-catenin signaling plays a disparate role in different phases of fracture repair: implications for therapy to improve bone healing. PLoS Med 4: e249. 17676991 10.1371/journal.pmed.0040249 PMC1950214

[14] Boland GM, Perkins G, Hall DJ, Tuan RS (2004) Wnt 3a promotes proliferation and suppresses osteogenic differentiation of adult human mesenchymal stem cells. J Cell Biochem 93: 1210–1230. 15486964 10.1002/jcb.20284

[15] Ling L, Nurcombe V, Cool SM (2009) Wnt signaling controls the fate of mesenchymal stem cells. Gene 433: 1–7. 19135507 10.1016/j.gene.2008.12.008

[16] Hill TP, Spater D, Taketo MM, Birchmeier W, Hartmann C (2005) Canonical Wnt/beta-catenin signaling prevents osteoblasts from differentiating into chondrocytes. Dev Cell 8: 727–738. 15866163 10.1016/j.devcel.2005.02.013

[17] Rodda SJ, McMahon AP (2006) Distinct roles for Hedgehog and canonical Wnt signaling in specification, differentiation and maintenance of osteoblast progenitors. Development 133: 3231–3244. 16854976 10.1242/dev.02480

[18] Li X, Grisanti M, Fan W, Asuncion FJ, Tan HL, et al (2011) Dickkopf-1 regulates bone formation in young growing rodents and upon traumatic injury. J Bone Miner Res 26: 2610–2621. 21773994 10.1002/jbmr.472

[19] Secreto FJ, Hoeppner LH, Westendorf JJ (2009) Wnt signaling during fracture repair. Curr Osteoporos Rep 7: 64–69. 19631031 10.1007/s11914-009-0012-5 PMC2972700

[20] Claes L, Augat P, Schorlemmer S, Konrads C, Ignatius A, et al (2008) Temporary distraction and compression of a diaphyseal osteotomy accelerates bone healing. J Orthop Res 26: 772–777. 18240329 10.1002/jor.20588

[21] Rontgen V, Blakytny R, Matthys R, Landauer M, Wehner T, et al (2010) Fracture healing in mice under controlled rigid and flexible conditions using an adjustable external fixator. J Orthop Res 28: 1456–1462. 20872581 10.1002/jor.21148

[22] Pfaffl MW (2001) A new mathematical model for relative quantification in real-time RT-PCR. Nucleic Acids Res 29: e45. 11328886 10.1093/nar/29.9.e45 PMC55695

[23] Zhang X, Siclari VA, Lan S, Zhu J, Koyama E, et al (2011) The critical role of the epidermal growth factor receptor in endochondral ossification. J Bone Miner Res 26: 2622–2633. 21887704 10.1002/jbmr.502 PMC3200483

[24] Mansukhani A, Ambrosetti D, Holmes G, Cornivelli L, Basilico C (2005) Sox2 induction by FGF and FGFR2 activating mutations inhibits Wnt signaling and osteoblast differentiation. J Cell Biol 168: 1065–1076. 15781477 10.1083/jcb.200409182 PMC2171836

[25] Mosig RA, Martignetti JA (2012) Loss of MMP-2 in osteoblasts upregulates osteopontin and bone sialoprotein expression in a circuit regulating bone homeostasis. Dis Model Mech. 10.1242/dmm.007914 PMC3597021 22917927

[26] Filanti C, Dickson GR, Di Martino D, Ulivi V, Sanguineti C, et al (2000) The expression of metalloproteinase-2, -9, and -14 and of tissue inhibitors-1 and -2 is developmentally modulated during osteogenesis in vitro, the mature osteoblastic phenotype expressing metalloproteinase-14. J Bone Miner Res 15: 2154–2168. 11092396 10.1359/jbmr.2000.15.11.2154

[27] Xu L, Zhang G, Zhou Y, Chen Y, Xu W, et al (2011) Stimulation of B7-H3 (CD276) directs the differentiation of human marrow stromal cells to osteoblasts. Immunobiology 216: 1311–1317. 21893365 10.1016/j.imbio.2011.05.013

[28] Glass DA 2nd, Bialek P, Ahn JD, Starbuck M, Patel MS, et al (2005) Canonical Wnt signaling in differentiated osteoblasts controls osteoclast differentiation. Dev Cell 8: 751–764. 15866165 10.1016/j.devcel.2005.02.017

[29] The HYP Consortium (1995) A gene (PEX) with homologies to endopeptidases is mutated in patients with X-linked hypophosphatemic rickets. Nat Genet 11: 130–136. 7550339 10.1038/ng1095-130

[30] Aubin I, Adams CP, Opsahl S, Septier D, Bishop CE, et al (2005) A deletion in the gene encoding sphingomyelin phosphodiesterase 3 (Smpd3) results in osteogenesis and dentinogenesis imperfecta in the mouse. Nat Genet 37: 803–805. 16025116 10.1038/ng1603

[31] Takeda H, Ozaki K, Yasuda H, Ishida M, Kitano S, et al (1998) Sphingomyelinase and ceramide inhibit formation of F-actin ring in and bone resorption by rabbit mature osteoclasts. FEBS Lett 422: 255–258. 9490019 10.1016/s0014-5793(98)00005-2

[32] Yoshimine Y, Tsukuba T, Isobe R, Sumi M, Akamine A, et al (1995) Specific immunocytochemical localization of cathepsin E at the ruffled border membrane of active osteoclasts. Cell Tissue Res 281: 85–91. 7621529 10.1007/BF00307961

[33] Li Y, Lacerda DA, Warman ML, Beier DR, Yoshioka H, et al (1995) A fibrillar collagen gene, Col11a1, is essential for skeletal morphogenesis. Cell 80: 423–430. 7859283 10.1016/0092-8674(95)90492-1

[34] Yadav VK, Ryu JH, Suda N, Tanaka KF, Gingrich JA, et al (2008) Lrp5 controls bone formation by inhibiting serotonin synthesis in the duodenum. Cell 135: 825–837. 19041748 10.1016/j.cell.2008.09.059 PMC2614332

[35] Cui Y, Niziolek PJ, MacDonald BT, Zylstra CR, Alenina N, et al (2011) Lrp5 functions in bone to regulate bone mass. Nat Med 17: 684–691. 21602802 10.1038/nm.2388 PMC3113461

[36] Kim KA, Wagle M, Tran K, Zhan X, Dixon MA, et al (2008) R-Spondin family members regulate the Wnt pathway by a common mechanism. Mol Biol Cell 19: 2588–2596. 18400942 10.1091/mbc.E08-02-0187 PMC2397303

[37] Zhong N, Gersch RP, Hadjiargyrou M (2006) Wnt signaling activation during bone regeneration and the role of Dishevelled in chondrocyte proliferation and differentiation. Bone 39: 5–16. 16459154 10.1016/j.bone.2005.12.008

[38] Iwaniec UT, Wronski TJ, Liu J, Rivera MF, Arzaga RR, et al (2007) PTH stimulates bone formation in mice deficient in Lrp5. J Bone Miner Res 22: 394–402. 17147489 10.1359/jbmr.061118

[39] Bollerslev J, Marks SC Jr, Pockwinse S, Kassem M, Brixen K, et al (1993) Ultrastructural investigations of bone resorptive cells in two types of autosomal dominant osteopetrosis. Bone 14: 865–869. 8155410 10.1016/8756-3282(93)90316-3

[40] Henriksen K, Gram J, Hoegh-Andersen P, Jemtland R, Ueland T, et al (2005) Osteoclasts from patients with autosomal dominant osteopetrosis type I caused by a T253I mutation in low-density lipoprotein receptor-related protein 5 are normal in vitro, but have decreased resorption capacity in vivo. Am J Pathol 167: 1341–1348. 16251418 10.1016/S0002-9440(10)61221-7 PMC1603785

[41] Pinson KI, Brennan J, Monkley S, Avery BJ, Skarnes WC (2000) An LDL-receptor-related protein mediates Wnt signalling in mice. Nature 407: 535–538. 11029008 10.1038/35035124

[42] Riddle RC, Diegel CR, Leslie JM, Van Koevering KK, Faugere MC, et al (2013) Lrp5 and Lrp6 exert overlapping functions in osteoblasts during postnatal bone acquisition. PLoS One 8: e63323. 23675479 10.1371/journal.pone.0063323 PMC3651091

[43] Holmen SL, Giambernardi TA, Zylstra CR, Buckner-Berghuis BD, Resau JH, et al (2004) Decreased BMD and limb deformities in mice carrying mutations in both Lrp5 and Lrp6. J Bone Miner Res 19: 2033–2040. 15537447 10.1359/JBMR.040907

[44] Holmen SL, Zylstra CR, Mukherjee A, Sigler RE, Faugere MC, et al (2005) Essential role of beta-catenin in postnatal bone acquisition. J Biol Chem 280: 21162–21168. 15802266 10.1074/jbc.M501900200

