# Supplementary material for: Osteoblast-Specific Krm2 Overexpression and Lrp5 Deficiency Have Different Effects on Fracture Healing in Mice
Source: PLoS One. 2014 Jul 25;9(7):e103250. doi: 10.1371/journal.pone.0103250 (PMC4111586; doi:10.1371/journal.pone.0103250)
Supplement: Table S1 — Differentially expressed gene products in fracture calli of Col1a1-Krm2 and Lrp5−/− mice. Differential gene expression in callus tissue under semi-rigid fixation was analyzed 10 days post-fracture by microarray analysis (n = 3 each). Only gene products that fulfilled criteria for significant gene expression changes (see methods) are listed. FC: mean value of fold changes of the cross-wise comparisons with wildtype callus. SD: standard deviation. ― no significantly differential expression when compared to wildtype callus. (PDF) [file pone.0103250.s001.pdf]

			Col1a1
Gene	Gene Title	Probe set ID	FC
Abcb10	ATP-binding cassette, sub-family B (MDR/TAP), m	1416402_at	2,52
Abcb10	ATP-binding cassette, sub-family B (MDR/TAP), m	1416403_at	2,62
Abi3bp	ABI gene family, member 3 (NESH) binding protei	1427053_at	0,72
Acap1	ArfGAP with coiled-coil, ankyrin repeat and PH do	1434873_a_at	2,97
Acsl1	acyl-CoA synthetase long-chain family member 1	1423883_at	2,02
Acsl1	acyl-CoA synthetase long-chain family member 1	1450643_s_at	2,18
Acsl6	acyl-CoA synthetase long-chain family member 6	1451257_at	2,05
Actn2	actinin alpha 2	1448327_at	2,12
Adam12	a disintegrin and metallopeptidase domain 12 (meltr	1421171_at	0,54
Adam12	a disintegrin and metallopeptidase domain 12 (meltr	1421172_at	0,61
Adamts15	a disintegrin-like and metallopeptidase (reprolysin t	1427056_at	2,98
Adamts4	a disintegrin-like and metallopeptidase (reprolysin t	1455965_at	0,49
Adamts9	a disintegrin-like and metallopeptidase (reprolysin t	1430352_at	0,23
Adamts9	a disintegrin-like and metallopeptidase (reprolysin t	1437785_at	0,59
Add2	adducin 2 (beta)	1435287_at	4,70
Adrbk2	adrenergic receptor kinase, beta 2	1440801_s_at	—
Aebp1	AE binding protein 1	1450637_a_at	0,59
Agfg2	ArfGAP with FG repeats 2	1425362_at	3,05
Ahsp	alpha hemoglobin stabilizing protein	1449077_at	3,03
AI256396 /// LOC100	EST AI256396 /// hypothetical LOC100503463	1457042_at	0,59
AI314760	expressed sequence AI314760	1437923_at	0,59
AI464131	expressed sequence AI464131	1435417_at	2,15
Akap9	A kinase (PRKA) anchor protein (yotiao) 9	1455151_at	—
Alad	aminolevulinate, delta-, dehydratase	1424877_a_at	6,14
Alas2	aminolevulinic acid synthase 2, erythroid	1451675_a_at	4,02
Aldh18a1	aldehyde dehydrogenase 18 family, member A1	1437325_x_at	0,73
Aldh1a1	aldehyde dehydrogenase family 1, subfamily A1	1416468_at	2,70
Aldh1a2	aldehyde dehydrogenase family 1, subfamily A2	1422789_at	2,26
Aldh1a3	aldehyde dehydrogenase family 1, subfamily A3	1448789_at	—
Aldh1l2	aldehyde dehydrogenase 1 family, member L2	1436119_at	0,66
Aldh2	aldehyde dehydrogenase 2, mitochondrial	1448143_at	1,71
Alpk2	alpha-kinase 2	1452478_at	2,05
Alpl	alkaline phosphatase, liver/bone/kidney	1423611_at	0,57
Amd1	S-adenosylmethionine decarboxylase 1	1416835_s_at	1,65
Ang	angiogenin, ribonuclease, RNase A family, 5	1438936_s_at	0,69
Angptl1	angiopoietin-like 1	1455224_at	0,57
Angptl2	angiopoietin-like 2	1455090_at	0,71
Angptl4	angiopoietin-like 4	1417130_s_at	0,62
Ank1	ankyrin 1, erythroid	1419421_at	2,18
Ankrd17	ankyrin repeat domain 17	1436775_a_at	—
Ankrd2	ankyrin repeat domain 2 (stretch responsive muscle)	1419621_at	3,93
Ankrd33b	ankyrin repeat domain 33B	1453782_at	2,46
Ankrd43	ankyrin repeat domain 43	1436998_at	27,26
Antxr1	anthrax toxin receptor 1	1451446_at	0,67
Anxa2	annexin A2	1419091_a_at	0,76
Anxa4	annexin A4	1421223_a_at	0,67
Anxa5	annexin A5	1425567_a_at	0,71
Ap1s3	adaptor-related protein complex AP-1, sigma 3	1455735_at	2,95
Ap2a2	adaptor protein complex AP-2, alpha 2 subunit	1432007_s_at	1,88
Ap3m1	adaptor-related protein complex 3, mu 1 subunit	1448308_at	0,79
Aplnr	apelin receptor	1438651_a_at	0,68

Apod	apolipoprotein D	1416371_at	0,60
Apol8	apolipoprotein L 8	1441054_at	3,65
Araf	v-raf murine sarcoma 3611 viral oncogene homolog	1440764_at	1,69
Arcn1	archain 1	1436062_at	0,63
Arhgap12	Rho GTPase activating protein 12	1451526_at	0,61
Arl1	ADP-ribosylation factor-like 1	1451025_at	0,71
Arrb1	arrestin, beta 1	1460444_at	2,07
Asb2	ankyrin repeat and SOCS box-containing 2	1428444_at	2,13
Asf1b	ASF1 anti-silencing function 1 homolog B (S. cerev	1423714_at	1,69
Ash1l	ash1 (absent, small, or homeotic)-like (Drosophila)	1450071_at	—
Aspn	asporin	1416652_at	0,55
Aspn	asporin	1448421_s_at	0,63
Atad2	ATPase family, AAA domain containing 2	1436174_at	1,58
Atox1	ATX1 (antioxidant protein 1) homolog 1 (yeast)	1415760_s_at	0,73
Atp1a2	ATPase, Na+/K+ transporting, alpha 2 polypeptide	1452308_a_at	2,63
Atp6v0d2	ATPase, H+ transporting, lysosomal V0 subunit D2	1434798_at	0,66
Atp6v0e	ATPase, H+ transporting, lysosomal V0 subunit E	1416328_a_at	0,77
Atp8a1	ATPase, aminophospholipid transporter (APLT), cla	1454728_s_at	1,78
Atp8a1	ATPase, aminophospholipid transporter (APLT), cla	1423597_at	2,30
Aurkb	aurora kinase B	1424128_x_at	1,63
B3galnt1	UDP-GalNAc:betaGlcNAc beta 1,3-galactosaminylt	1418736_at	0,70
B3galnt2	UDP-GalNAc:betaGlcNAc beta 1,3-galactosaminylt	1454842_a_at	1,49
B4galt1	UDP-Gal:betaGlcNAc beta 1,4- galactosyltransferas	1418014_a_at	0,78
Bambi	BMP and activin membrane-bound inhibitor, homolo	1423753_at	0,58
Baz1a /// LOC100505	bromodomain adjacent to zinc finger domain 1A ///	1447930_at	1,78
BC005512 /// F63000	cDNA sequence BC005512 /// RIKEN cDNA F630C	1426936_at	—
BC018101 /// Zfp97	cDNA sequence BC018101 /// zinc finger protein 97	1449972_s_at	0,59
BC094435	cDNA sequence BC094435	1455316_x_at	1,50
BC151093 /// Ear1 ///	cDNA sequence BC151093 /// eosinophil-associated	1422411_s_at	—
Bcl11a /// LOC10050	B-cell CLL/lymphoma 11A (zinc finger protein) ///	1456632_at	3,38
Bcl11a /// LOC10050	B-cell CLL/lymphoma 11A (zinc finger protein) ///	1457072_at	3,51
Bcl6	B-cell leukemia/lymphoma 6	1421818_at	1,56
Bet1	blocked early in transport 1 homolog (S. cerevisiae)	1416866_at	0,69
Bicc1	bicaudal C homolog 1 (Drosophila)	1423484_at	0,47
Blvrb	biliverdin reductase B (flavin reductase (NADPH))	1451386_at	1,93
Bnip3l	BCL2/adenovirus E1B interacting protein 3-like	1416922_a_at	1,34
Bok	BCL2-related ovarian killer protein	1417040_a_at	0,70
Bpgm	2,3-bisphosphoglycerate mutase	1415864_at	2,84
Bpgm	2,3-bisphosphoglycerate mutase	1415865_s_at	3,02
Bpgm	2,3-bisphosphoglycerate mutase	1448119_at	3,21
Bsdc1	BSD domain containing 1	1427270_a_at	1,74
Btg2	B-cell translocation gene 2, anti-proliferative	1416250_at	2,12
C1qtnf5 /// Mfrp	C1q and tumor necrosis factor related protein 5 /// m	1424762_at	0,70
C1qtnf6	C1q and tumor necrosis factor related protein 6	1431856_a_at	0,58
C730029A08Rik	RIKEN cDNA C730029A08 gene	1436168_at	2,93
Cacna2d1	calcium channel, voltage-dependent, alpha2/delta su	1449999_a_at	2,86
Cald1	caldesmon 1	1424769_s_at	0,71
Calu	calumenin	1415870_at	0,67
Camk4	calcium/calmodulin-dependent protein kinase IV	1439843_at	0,48
Car1	carbonic anhydrase 1	1416193_at	4,14
Car2	carbonic anhydrase 2	1448752_at	2,97
Ccdc80	coiled-coil domain containing 80	1424187_at	0,60
Ccl9	chemokine (C-C motif) ligand 9	1448898_at	0,60
Ccl9	chemokine (C-C motif) ligand 9	1417936_at	0,63
Ccna2	cyclin A2	1417910_at	1,53
Ccnb2	cyclin B2	1450920_at	1,72

Ccndbp1	cyclin D-type binding-protein 1	1420745_a_at	1,37
Ccne2	cyclin E2	1422535_at	1,76
Ccrn4l	CCR4 carbon catabolite repression 4-like (S. cerevis	1425837_a_at	1,61
Cd109	CD109 antigen	1425658_at	0,64
Cd24a	CD24a antigen	1437502_x_at	1,83
Cd24a	CD24a antigen	1448182_a_at	2,09
Cd24a	CD24a antigen	1416034_at	2,55
Cd276	CD276 antigen	1417599_at	0,58
Cd34	CD34 antigen	1416072_at	0,66
Cd5l	CD5 antigen-like	1449193_at	2,26
Cdc25b	cell division cycle 25 homolog B (S. pombe)	1421963_a_at	1,93
Cdc26	cell division cycle 26	1423987_at	0,69
Cdc42ep1	CDC42 effector protein (Rho GTPase binding) 1	1424376_at	0,59
Cdc6	cell division cycle 6 homolog (S. cerevisiae)	1417019_a_at	3,03
Cdca8	cell division cycle associated 8	1428481_s_at	1,63
Cdca8	cell division cycle associated 8	1436847_s_at	1,75
Cdh11	cadherin 11	1450757_at	0,71
Cdh2	cadherin 2	1418815_at	0,63
Cdk4	cyclin-dependent kinase 4	1422440_at	0,66
Cdr2	cerebellar degeneration-related 2	1417430_at	1,68
Cdt1	chromatin licensing and DNA replication factor 1	1424143_a_at	1,62
Cenpa	centromere protein A	1450842_a_at	1,51
Ces2g	carboxylesterase 2G	1424968_at	2,45
Cfd	complement factor D (adipsin)	1417867_at	7,97
Cfdp1	craniofacial development protein 1	1416742_at	0,70
Cgref1	cell growth regulator with EF hand domain 1	1424529_s_at	0,45
Cgref1	cell growth regulator with EF hand domain 1	1424528_at	0,47
Chac2	ChaC, cation transport regulator homolog 2 (E. coli)	1419074_at	1,99
Chd4	chromodomain helicase DNA binding protein 4	1436343_at	—
Chrdl1	chordin-like 1	1434201_at	0,63
Clcn3	chloride channel 3	1433486_at	1,95
Clcn3	chloride channel 3	1438366_x_at	2,06
Cldn13	claudin 13	1422920_at	3,88
Clip1	CAP-GLY domain containing linker protein 1	1431098_at	2,17
Clns1a	chloride channel, nucleotide-sensitive, 1A	1423181_s_at	0,76
Cnn3	calponin 3, acidic	1426724_at	0,72
Cnot7	CCR4-NOT transcription complex, subunit 7	1430519_a_at	2,35
Cnpy2	canopy 2 homolog (zebrafish)	1437783_x_at	0,58
Cnpy2	canopy 2 homolog (zebrafish)	1448372_a_at	0,63
Cnpy4	canopy 4 homolog (zebrafish)	1428325_at	0,63
Col11a1	collagen, type XI, alpha 1	1449154_at	—
Col12a1	collagen, type XII, alpha 1	1427391_a_at	0,60
Col13a1	collagen, type XIII, alpha 1	1422866_at	0,61
Col16a1	collagen, type XVI, alpha 1	1427986_a_at	0,64
Col19a1	collagen, type XIX, alpha 1	1456953_at	2,36
Col1a2	collagen, type I, alpha 2	1446326_at	0,66
Col22a1	collagen, type XXII, alpha 1	1453084_s_at	0,56
Col5a1	collagen, type V, alpha 1	1416741_at	0,60
Col8a2	collagen, type VIII, alpha 2	1434667_at	0,61
Copb2	coatomer protein complex, subunit beta 2 (beta prim	1456175_a_at	0,64
Copb2	coatomer protein complex, subunit beta 2 (beta prim	1452102_at	0,70
Cops2	COP9 (constitutive photomorphogenic) homolog, su	1423459_at	0,77
Coq10a	coenzyme Q10 homolog A (yeast)	1433628_at	2,14
Coq5	coenzyme Q5 homolog, methyltransferase (yeast)	1417264_at	1,62
Cpeb3	cytoplasmic polyadenylation element binding protei	1455372_at	1,48
Cpeb3	cytoplasmic polyadenylation element binding protei	1437765_at	2,33

Cpox	coproporphyrinogen oxidase	1422492_at	2,28
Cpxm2	carboxypeptidase X 2 (M14 family)	1460248_at	—
Creb3	cAMP responsive element binding protein 3	1419979_s_at	0,57
Creb3	cAMP responsive element binding protein 3	1424740_at	0,68
Crebbp	CREB binding protein	1436983_at	—
Creg1	cellular repressor of E1A-stimulated genes 1	1415947_at	1,49
Crip1	cysteine-rich protein 1 (intestinal)	1416326_at	0,78
Crispld2	cysteine-rich secretory protein LCCL domain contai	1437056_x_at	—
Csnk1a1	casein kinase 1, alpha 1	1430529_at	—
Cspp1	centrosome and spindle pole associated protein 1	1431405_a_at	—
Csprs /// Gm7592	component of Sp100-rs /// predicted gene 7592	1435792_at	7,24
Csrp1	cysteine and glycine-rich protein 1	1425811_a_at	0,59
Csrp1	cysteine and glycine-rich protein 1	1425810_a_at	0,60
Csrp2	cysteine and glycine-rich protein 2	1420731_a_at	0,58
Ctcf	CCCTC-binding factor	1449042_at	1,80
Cthrc1	collagen triple helix repeat containing 1	1452968_at	0,59
Ctse	cathepsin E	1418989_at	4,22
Cux1	Cut-like homeobox 1	1441956_s_at	1,77
Cxcr4	chemokine (C-X-C motif) receptor 4	1448710_at	3,01
Cyb5r3	cytochrome b5 reductase 3	1422185_a_at	0,68
Cyb5r3	cytochrome b5 reductase 3	1422186_s_at	0,75
Cyfip2	cytoplasmic FMR1 interacting protein 2	1449273_at	2,13
D17Wsu104e	DNA segment, Chr 17, Wayne State University 104,	1416696_at	0,63
D4Bwg0951e	DNA segment, Chr 4, Brigham & Women's Genetics	1428384_at	0,69
D930015E06Rik	RIKEN cDNA D930015E06 gene	1426834_s_at	2,38
Dad1	defender against cell death 1	1454860_x_at	0,67
Dbp	D site albumin promoter binding protein	1418174_at	—
Dbp	D site albumin promoter binding protein	1438211_s_at	—
Dck	deoxycytidine kinase	1449176_a_at	1,74
Dctn3	dynactin 3	1416247_at	0,64
Ddah1	dimethylarginine dimethylaminohydrolase 1	1454995_at	0,66
Ddah1	dimethylarginine dimethylaminohydrolase 1	1455400_at	0,66
Ddi2	DNA-damage inducible protein 2	1429093_at	1,66
Dhrs11	dehydrogenase/reductase (SDR family) member 11	1425704_at	2,85
Dkk3	dickkopf homolog 3 (Xenopus laevis)	1417312_at	0,50
Dkk3	dickkopf homolog 3 (Xenopus laevis)	1448669_at	0,53
Dna2	DNA replication helicase 2 homolog (yeast)	1452210_at	1,83
Dnajb6	DnaJ (Hsp40) homolog, subfamily B, member 6	1434035_at	1,66
Dnajc3	DnaJ (Hsp40) homolog, subfamily C, member 3	1419162_s_at	0,67
Dnmt1	DNA methyltransferase (cytosine-5) 1	1435122_x_at	1,68
Dpt	dermatopontin	1418511_at	0,67
Dpysl3	dihydropyrimidinase-like 3	1454613_at	0,70
Dsel	dermatan sulfate epimerase-like	1438407_at	0,62
Dvl1	dishevelled, dsh homolog 1 (Drosophila)	1437301_a_at	1,44
Dynlrb1	dynein light chain roadblock-type 1	1428257_s_at	0,74
Dysfip1	dysferlin interacting protein 1	1431281_at	2,59
E2f2	E2F transcription factor 2	1436434_at	2,99
E2f2	E2F transcription factor 2	1455790_at	3,01
E2f7	E2F transcription factor 7	1437187_at	1,70
E2f8	E2F transcription factor 8	1436186_at	2,45
Ebf1	early B-cell factor 1	1448293_at	0,52
Ebf1	early B-cell factor 1	1416301_a_at	0,68
Edil3	EGF-like repeats and discoidin I-like domains 3	1433474_at	0,60
Egfr	epidermal growth factor receptor	1435888_at	0,62
Egln3	EGL nine homolog 3 (C. elegans)	1418649_at	1,70
Ehbp1l1	EH domain binding protein 1-like 1	1448467_a_at	1,60

Eif1a	eukaryotic translation initiation factor 1A	1424344_s_at	0,72
Eif2c2	eukaryotic translation initiation factor 2C, 2	1426366_at	2,40
Eif3a	eukaryotic translation initiation factor 3, subunit A	1416661_at	1,86
Eif3c	eukaryotic translation initiation factor 3, subunit C	1415859_at	2,00
Eif4a1	eukaryotic translation initiation factor 4A1	1430980_a_at	0,75
Eif4ebp2	eukaryotic translation initiation factor 4E binding pr	1417084_at	2,89
Eif5	eukaryotic translation initiation factor 5	1454664_a_at	1,46
Elp3	elongation protein 3 homolog (S. cerevisiae)	1426643_at	0,74
Emp3	epithelial membrane protein 3	1417104_at	0,76
Enpp4	ectonucleotide pyrophosphatase/phosphodiesterase 4	1434580_at	1,51
Entpd4	ectonucleoside triphosphate diphosphohydrolase 4	1449190_a_at	0,49
Entpd4	ectonucleoside triphosphate diphosphohydrolase 4	1438177_x_at	0,53
Entpd4	ectonucleoside triphosphate diphosphohydrolase 4	1447900_x_at	0,54
Epb4.1	erythrocyte protein band 4.1	1424092_at	3,17
Epb4.1l3	erythrocyte protein band 4.1-like 3	1419062_at	0,68
Epb4.2	erythrocyte protein band 4.2	1417337_at	5,19
Ermap	erythroblast membrane-associated protein	1418909_at	4,70
Esco2	establishment of cohesion 1 homolog 2 (S. cerevisia	1428304_at	1,99
Exoc6	exocyst complex component 6	1460576_at	1,54
Ezh2	enhancer of zeste homolog 2 (Drosophila)	1416544_at	1,59
Ezr	ezrin	1450850_at	1,74
Fam114a1	family with sequence similarity 114, member A1	1417272_at	0,62
Fam114a1	family with sequence similarity 114, member A1	1448648_at	0,62
Fam117a	family with sequence similarity 117, memberA	1433639_at	2,81
Fam126b	family with sequence similarity 126, member B	1456257_at	0,47
Fam129c	family with sequence similarity 129, member C	1457728_at	4,07
Fam167a	family with sequence similarity 167, member A	1455872_at	0,40
Fam168a	family with sequence similarity 168, member A	1434375_at	0,36
Fam195b	family with sequence similarity 195, member B	1460690_at	0,73
Fam198b	family with sequence similarity 198, member B	1416805_at	0,70
Fam46c	family with sequence similarity 46, member C	1429682_at	3,48
Fam53b	family with sequence similarity 53, member B	1455428_at	1,75
Fam63b	family with sequence similarity 63, member B	1436842_at	1,47
Fam92a	family with sequence similarity 92, member A	1451570_a_at	0,67
Fat4	FAT tumor suppressor homolog 4 (Drosophila)	1459749_s_at	0,64
Fbn1	fibrillin 1	1425896_a_at	0,67
Fbn2	fibrillin 2	1454830_at	0,47
Fbn2	fibrillin 2	1422831_at	—
Fbxo32	F-box protein 32	1448747_at	2,48
Fbxo5	F-box protein 5	1429499_at	2,19
Fbxo9	f-box protein 9	1417480_at	1,61
Fbxo9	f-box protein 9	1432211_a_at	1,77
Fcho2	FCH domain only 2	1442453_at	3,54
Fech	ferrochelatase	1418699_s_at	1,87
Fech	ferrochelatase	1418698_a_at	2,07
Fen1	flap structure specific endonuclease 1	1421731_a_at	1,74
Fen1	flap structure specific endonuclease 1	1436454_x_at	1,78
Fgf13	fibroblast growth factor 13	1418497_at	1,88
Fgfr2	fibroblast growth factor receptor 2	1433489_s_at	0,61
Fhdc1	FH2 domain containing 1	1439018_at	2,41
Fhdc1	FH2 domain containing 1	1443657_at	3,02
Fignl1	fidgetin-like 1	1422430_at	1,85
Fkbp10	FK506 binding protein 10	1449632_s_at	0,49
Fkbp10	FK506 binding protein 10	1415951_at	0,53
Fkbp14	FK506 binding protein 14	1433481_at	0,59
Fkbp7	FK506 binding protein 7	1416803_at	0,66

Fmod	fibromodulin	1437718_x_at	—
Fnbp4	formin binding protein 4	1438700_at	2,99
Fndc1	fibronectin type III domain containing 1	1453321_at	0,60
Frg1	FSHD region gene 1	1417253_at	1,69
Fry	furry homolog (Drosophila)	1456480_at	1,66
Fubp1	far upstream element (FUSE) binding protein 1	1437544_at	—
Fus	fusion, derived from t(12	1455831_at	1,81
Fxn	frataxin	1427282_a_at	1,86
Fyco1	FYVE and coiled-coil domain containing 1	1427177_at	1,66
Gadd45a	growth arrest and DNA-damage-inducible 45 alpha	1449519_at	2,26
Gapvd1	GTPase activating protein and VPS9 domains 1	1453146_at	1,78
Gas2l3	growth arrest-specific 2 like 3	1455980_a_at	—
Gas2l3	growth arrest-specific 2 like 3	1437244_at	—
Gbp8	guanylate-binding protein 8	1418776_at	2,92
Gca	grancalcin	1424698_s_at	2,20
Gch1	GTP cyclohydrolase 1	1429692_s_at	2,39
Gch1	GTP cyclohydrolase 1	1420499_at	2,80
Gclc	glutamate-cysteine ligase, catalytic subunit	1424296_at	2,11
Gclm	glutamate-cysteine ligase, modifier subunit	1418627_at	2,20
Gfi1b	growth factor independent 1B	1420399_at	3,86
Glo1	glyoxalase 1	1424108_at	1,53
Glo1	glyoxalase 1	1424109_a_at	1,53
Glrx5	glutaredoxin 5 homolog (S. cerevisiae)	1428553_at	1,58
Glt25d1	glycosyltransferase 25 domain containing 1	1433496_at	0,47
Glt25d1	glycosyltransferase 25 domain containing 1	1433495_at	0,55
Glt8d1	glycosyltransferase 8 domain containing 1	1428269_a_at	0,73
Gm10393 /// Plac9	predicted gene 10393 /// placenta specific 9	1452590_a_at	0,53
Gm10883	predicted gene 10883	1452463_x_at	1,94
Gm10883 /// Gm1420	predicted gene 10883 /// predicted gene 1420 /// pred	1427455_x_at	1,77
Gm11276 /// Hist1h2a	predicted gene 11276 /// histone cluster 1, H2ao	1438009_at	1,56
Gm14085 /// Slc28a2	predicted gene 14085 /// solute carrier family 28 (so	1450639_at	2,61
Gm4354	predicted gene 4354	1424609_a_at	1,90
Gm4354	predicted gene 4354	1424607_a_at	1,96
Gm5526 /// Gm8894 /	predicted pseudogene 5526 /// predicted gene 8894 /	1424269_a_at	0,80
Gm6453 /// Hmbs	predicted gene 6453 /// hydroxymethylbilane syntha	1436930_x_at	2,68
Gm9222	predicted gene 9222	1438050_x_at	1,78
Gna13	guanine nucleotide binding protein, alpha 13	1422556_at	1,96
Gng11	guanine nucleotide binding protein (G protein), gam	1448942_at	0,70
Gnpda2	glucosamine-6-phosphate deaminase 2	1426523_a_at	0,63
Gnpnat1	glucosamine-phosphate N-acetyltransferase 1	1423156_at	0,61
Golga4	golgi autoantigen, golgin subfamily a, 4	1448803_at	1,69
Gp1ba	glycoprotein 1b, alpha polypeptide	1422316_at	2,52
Gpam	glycerol-3-phosphate acyltransferase, mitochondrial	1419499_at	1,79
Gpc1	glypican 1	1417389_at	0,66
Gpd2	glycerol phosphate dehydrogenase 2, mitochondrial	1417434_at	1,52
Gpt2	glutamic pyruvate transaminase (alanine aminotrans	1438385_s_at	1,63
Gpt2	glutamic pyruvate transaminase (alanine aminotrans	1455007_s_at	1,89
Gpx1	glutathione peroxidase 1	1460671_at	1,26
Gpx3	glutathione peroxidase 3	1449106_at	0,61
Gpx7	glutathione peroxidase 7	1417836_at	0,61
Gpx8	glutathione peroxidase 8 (putative)	1424099_at	0,58
Grina	glutamate receptor, ionotropic, N-methyl D-aspartat	1436297_a_at	1,72
Grina	glutamate receptor, ionotropic, N-methyl D-aspartat	1417423_at	1,86
Gulp1	GULP, engulfment adaptor PTB domain containing	1453771_at	0,59
Gulp1	GULP, engulfment adaptor PTB domain containing	1434423_at	0,62
Gypa	glycophorin A	1423016_a_at	3,94

Gypa	glycophorin A	1425643_at	4,96
Gypc	glycophorin C	1423878_at	2,00
Gys1	glycogen synthase 1, muscle	1416737_at	2,03
H2-Aa	histocompatibility 2, class II antigen A, alpha	1443783_x_at	0,15
H2afx	H2A histone family, member X	1416746_at	1,65
H2-D1	histocompatibility 2, D region locus 1	1451683_x_at	0,21
H2-D1	histocompatibility 2, D region locus 1	1450170_x_at	62,61
H2-DMb2	histocompatibility 2, class II, locus Mb2	1443687_x_at	2,35
H2-Ea-ps /// LOC100	histocompatibility 2, class II antigen E alpha, pseud	1422892_s_at	74,06
H2-Q7	histocompatibility 2, Q region locus 7	1418536_at	0,44
Hagh	hydroxyacyl glutathione hydrolase	1424171_a_at	1,80
Hddc3	HD domain containing 3	1428692_at	3,25
Hdlbp	high density lipoprotein (HDL) binding protein	1415987_at	0,66
Hebp1	heme binding protein 1	1418172_at	1,85
Hemgn	hemogen	1418199_at	4,16
Hipk2	homeodomain interacting protein kinase 2	1428433_at	1,50
Hk2	hexokinase 2	1422612_at	1,87
Hmbs	hydroxymethylbilane synthase	1426475_at	2,77
Hmcn1	hemicentin 1	1438532_at	0,49
Hmgb2	high mobility group box 2	1452534_a_at	1,81
Hmgn3	high mobility group nucleosomal binding domain 3	1431777_a_at	0,63
Hmgn3	high mobility group nucleosomal binding domain 3	1434875_a_at	0,65
Hspb6	heat shock protein, alpha-crystallin-related, B6	1436332_at	2,10
Htra1	HtrA serine peptidase 1	1438251_x_at	0,65
Htra1	HtrA serine peptidase 1	1416749_at	0,65
Htra3	HtrA serine peptidase 3	1427029_at	0,51
Icam4	intercellular adhesion molecule 4, Landsteiner-Wien	1422930_at	4,19
Ifit3	interferon-induced protein with tetratricopeptide rep	1449025_at	0,59
Ifitm5	interferon induced transmembrane protein 5	1440216_at	0,45
Ifnar1	interferon (alpha and beta) receptor 1	1442222_at	2,08
Ifnz	interferon zeta	1427216_at	2,81
Ift20	intraflagellar transport 20 homolog (Chlamydomona	1451412_a_at	0,60
Ift27	intraflagellar transport 27 homolog (Chlamydomona	1434299_x_at	0,60
Igfbp4	insulin-like growth factor binding protein 4	1421992_a_at	0,63
Igfbp6	insulin-like growth factor binding protein 6	1417933_at	0,49
Igh-2 /// Igh-VJ558 ///	immunoglobulin heavy chain 2 (serum IgA) /// imm	1425763_x_at	2,62
Igh-2 /// Igh-VJ558 ///	immunoglobulin heavy chain 2 (serum IgA) /// imm	1429381_x_at	2,62
Igh-2 /// Igh-VJ558 ///	immunoglobulin heavy chain 2 (serum IgA) /// imm	1421653_a_at	2,62
Igh-3 /// Ighg	immunoglobulin heavy chain 3 (serum IgG2b) /// In	1426174_s_at	3,85
Igh-6	Immunoglobulin heavy chain 6 (heavy chain of IgM	1425385_a_at	4,78
Igh-6	Immunoglobulin heavy chain 6 (heavy chain of IgM	1427870_x_at	5,95
Igh-6	Immunoglobulin heavy chain 6 (heavy chain of IgM	1427756_x_at	14,21
Igh-6	Immunoglobulin heavy chain 6 (heavy chain of IgM	1425324_x_at	14,55
Ighg	Immunoglobulin heavy chain (gamma polypeptide)	1424631_a_at	3,45
Ighg1	immunoglobulin heavy constant gamma 1 (G1m ma	1425247_a_at	10,81
Igj	immunoglobulin joining chain	1424305_at	2,46
Igl-C1 /// Igl-V1 /// L	immunoglobulin lambda chain, constant region 1 ///	1424931_s_at	4,16
Igl-V1	immunoglobulin lambda chain, variable 1	1430523_s_at	4,08
Ikbip	IKBKB interacting protein	1429065_at	0,59
Il13ra1	interleukin 13 receptor, alpha 1	1454783_at	0,69
Il1rl1	interleukin 1 receptor-like 1	1425145_at	2,64
Il2rg	interleukin 2 receptor, gamma chain	1416295_a_at	0,75
Il6st	interleukin 6 signal transducer	1437303_at	2,23
Il7r	interleukin 7 receptor	1448576_at	2,46
Ing5	inhibitor of growth family, member 5	1442094_at	2,78
Irf8	interferon regulatory factor 8	1448452_at	1,84

Isca1	iron-sulfur cluster assembly 1 homolog (S. cerevisia	1423651_at	1,51
Isg20	interferon-stimulated protein	1419569_a_at	2,19
Islr	immunoglobulin superfamily containing leucine-ricl	1418450_at	0,60
Itch	itchy, E3 ubiquitin protein ligase	1459332_at	—
Itgb1	integrin beta 1 (fibronectin receptor beta)	1426918_at	0,64
Itgbl1	integrin, beta-like 1	1425039_at	0,60
Itsn1	intersectin 1 (SH3 domain protein 1A)	1452338_s_at	—
Itsn2	intersectin 2	1423184_at	—
Jak1	Janus kinase 1	1433804_at	—
Jam2	junction adhesion molecule 2	1436568_at	0,66
Jkamp	JNK1/MAPK8-associated membrane protein	1441959_s_at	0,73
Kazald1	Kazal-type serine peptidase inhibitor domain 1	1436528_at	0,57
Kcnk1	potassium channel, subfamily K, member 1	1448690_at	0,54
Kcnma1	potassium large conductance calcium-activated chan	1428948_at	0,61
Kdelr2	KDEL (Lys-Asp-Glu-Leu) endoplasmic reticulum p	1417205_at	0,68
Kdelr3	KDEL (Lys-Asp-Glu-Leu) endoplasmic reticulum p	1418538_at	0,60
Kdm6a	4lysine (K)-specific demethylase 6A	1427672_a_at	1,57
Kel	Kell blood group	1449057_at	5,15
Kif1b	kinesin family member 1B	1425270_at	1,59
Kif1b	kinesin family member 1B	1451642_at	2,59
Kif5b	Kinesin family member 5B	1453524_at	1,87
Klf1	Kruppel-like factor 1 (erythroid)	1418600_at	5,57
Krt18	keratin 18	1448169_at	4,37
Krt8	keratin 8	1420647_a_at	4,42
Krtcap2	keratinocyte associated protein 2	1417058_a_at	0,63
Lamb1	laminin B1	1424113_at	0,58
Lamb1	laminin B1	1424114_s_at	0,68
Lbr	lamin B receptor	1415829_at	1,61
Ldb3	LIM domain binding 3	1451999_at	2,25
Lepre1	leprecan 1	1421462_a_at	0,57
Leprot	leptin receptor overlapping transcript	1451350_a_at	0,59
Lhfpl2	lipoma HMGIC fusion partner-like 2	1434129_s_at	—
Lmo2	LIM domain only 2	1454086_a_at	1,81
LOC100044751	hypothetical LOC100044751	1434752_at	2,06
LOC100503670	60S ribosomal protein L5-like	1451077_at	0,66
LOC100503670	60S ribosomal protein L5-like	1423666_s_at	0,72
LOC100504012 /// Lu	hypothetical LOC100504012 /// LUC7-like 2 (S. cer	1436766_at	—
LOC100505088	hypothetical LOC100505088	1423071_x_at	0,69
LOC100505359 /// Xl	x-linked lymphocyte-regulated protein 3A-like /// X-	1449347_a_at	2,23
Lox	lysyl oxidase	1448228_at	0,62
Lox	lysyl oxidase	1416121_at	0,68
Loxl1	lysyl oxidase-like 1	1451978_at	0,67
Lpar4	lysophosphatidic acid receptor 4	1439665_at	—
Lpgat1	lysophosphatidylglycerol acyltransferase 1	1424349_a_at	—
Lpin2	lipin 2	1452836_at	1,39
Lpp	LIM domain containing preferred translocation partn	1455314_at	0,69
Lrpap1	low density lipoprotein receptor-related protein asso	1426696_at	0,68
Lrrc15	leucine rich repeat containing 15	1436055_at	0,43
Lrrc15	leucine rich repeat containing 15	1453214_at	0,46
Lrrc17	leucine rich repeat containing 17	1429679_at	0,62
Lrrc59	leucine rich repeat containing 59	1416234_at	0,69
Lsp1	lymphocyte specific 1	1417756_a_at	0,61
Luc7l2	LUC7-like 2 (S. cerevisiae)	1436165_at	1,42
Luc7l2	LUC7-like 2 (S. cerevisiae)	1436767_at	—
Luc7l3	LUC7-like 3 (S. cerevisiae)	1451485_at	1,48
Ly6a	lymphocyte antigen 6 complex, locus A	1417185_at	0,61

Lyl1	lymphoblastomic leukemia 1	1419120_at	2,21
Maf	avian musculoaponeurotic fibrosarcoma (v-maf) AS	1437473_at	—
Mafg	v-maf musculoaponeurotic fibrosarcoma oncogene f	1448916_at	1,57
Mageb16	melanoma antigen family B, 16	1429701_at	9,65
Maged2	melanoma antigen, family D, 2	1426306_a_at	0,65
Malat1	metastasis associated lung adenocarcinoma transcrip	1429060_at	1,85
Malt1	mucosa associated lymphoid tissue lymphoma transl	1456429_at	1,89
Mamdc2	MAM domain containing 2	1453152_at	0,56
Man1a	mannosidase 1, alpha	1417111_at	0,75
March2	membrane-associated ring finger (C3HC4) 2	1459871_x_at	1,52
March3	membrane-associated ring finger (C3HC4) 3	1441643_at	4,64
Marcks	myristoylated alanine rich protein kinase C substrate	1415972_at	0,74
Marcks	myristoylated alanine rich protein kinase C substrate	1456028_x_at	0,77
Marcks	myristoylated alanine rich protein kinase C substrate	1415973_at	0,78
Marcksl1	MARCKS-like 1	1437226_x_at	0,70
Matn2	matrilin 2	1419442_at	0,48
Matn2	matrilin 2	1455978_a_at	0,53
Matr3	matrin 3	1458508_at	2,09
Mcart1	mitochondrial carrier triple repeat 1	1427425_at	2,17
Mcfd2	multiple coagulation factor deficiency 2	1424024_at	0,64
Mcm2	minichromosome maintenance deficient 2 mitotin (S	1434079_s_at	1,75
Mcm2	minichromosome maintenance deficient 2 mitotin (S	1448777_at	1,76
Mcm4	minichromosome maintenance deficient 4 homolog	1416214_at	1,60
Mdk	midkine	1416006_at	0,57
Med30	mediator complex subunit 30	1448917_at	1,38
Meox2	mesenchyme homeobox 2	1424233_at	0,62
Meox2	mesenchyme homeobox 2	1424234_s_at	0,65
Metrnl	meteorin, glial cell differentiation regulator-like	1424356_a_at	0,70
Mex3b	mex3 homolog B (C. elegans)	1437152_at	0,61
Mfap2	microfibrillar-associated protein 2	1417359_at	0,68
Mfap4	microfibrillar-associated protein 4	1424010_at	0,50
Mfap5	microfibrillar associated protein 5	1449082_at	0,42
Mfap5	microfibrillar associated protein 5	1418454_at	0,48
Mfn1	mitofusin 1	1426773_at	1,66
Mfsd4	major facilitator superfamily domain containing 4	1455531_at	2,63
Mid1	midline 1	1438239_at	0,37
Mier3	mesoderm induction early response 1, family memb	1460614_at	1,55
Mki67	antigen identified by monoclonal antibody Ki 67	1426817_at	1,78
Mknk2	MAP kinase-interacting serine/threonine kinase 2	1418300_a_at	1,62
Mkrn1	makorin, ring finger protein, 1	1434853_x_at	1,97
Mkrn1	makorin, ring finger protein, 1	1418434_at	2,06
Mkrn1	makorin, ring finger protein, 1	1455504_a_at	2,23
Mkrn1	makorin, ring finger protein, 1	1451425_a_at	2,64
Mkrn1	makorin, ring finger protein, 1	1418435_at	2,76
Mmp14	matrix metallopeptidase 14 (membrane-inserted)	1416572_at	0,54
Mmp2	matrix metallopeptidase 2	1439364_a_at	0,63
Mmp2	matrix metallopeptidase 2	1416136_at	0,64
Mphosph9	M-phase phosphoprotein 9	1431053_at	2,94
Mpo	myeloperoxidase	1415960_at	2,41
Mpzl1	myelin protein zero-like 1	1428168_at	0,66
Mpzl1	myelin protein zero-like 1	1428167_a_at	0,68
Msn	moesin	1421814_at	0,77
Mtap4	microtubule-associated protein 4	1443702_at	2,50
Mustn1	musculoskeletal, embryonic nuclear protein 1	1427201_at	—
Mxra7	matrix-remodelling associated 7	1440975_at	0,70
Mxra8	matrix-remodelling associated 8	1452330_a_at	0,66

Myb	myeloblastosis oncogene	1421317_x_at	3,21
Myb	myeloblastosis oncogene	1450194_a_at	3,56
Myb	myeloblastosis oncogene	1422734_a_at	4,33
Myh4	myosin, heavy polypeptide 4, skeletal muscle	1458368_at	3,08
Myl9	myosin, light polypeptide 9, regulatory	1452670_at	0,68
Myo1b	myosin IB	1447364_x_at	0,53
Myo1b	myosin IB	1459679_s_at	0,56
Myo1b	myosin IB	1448989_a_at	0,59
Myo1b	myosin IB	1427450_x_at	0,61
Myo1b	myosin IB	1448990_a_at	0,68
Mypn	myopalladin	1435813_at	2,87
Naa15	N(alpha)-acetyltransferase 15, NatA auxiliary subun	1418024_at	1,79
Ncam1	neural cell adhesion molecule 1	1426864_a_at	0,60
Ncapg2	non-SMC condensin II complex, subunit G2	1417926_at	1,74
Ncaph	non-SMC condensin I complex, subunit H	1436707_x_at	1,56
Ncf4	neutrophil cytosolic factor 4	1418465_at	1,52
Ndrg4	N-myc downstream regulated gene 4	1436188_a_at	0,53
Ndrg4	N-myc downstream regulated gene 4	1426615_s_at	0,59
Neat1	nuclear paraspeckle assembly transcript 1 (non-prot	1428083_at	1,77
Nedd4	neural precursor cell expressed, developmentally do	1421955_a_at	0,61
Neo1	neogenin	1447693_s_at	0,68
Ngrn	neugrin, neurite outgrowth associated	1416709_a_at	1,55
Nhp2	NHP2 ribonucleoprotein homolog (yeast)	1416605_at	0,67
Nipa1	non imprinted in Prader-Willi/Angelman syndrome	1434864_at	1,57
Nnmt	nicotinamide N-methyltransferase	1432517_a_at	0,47
Nnt	nicotinamide nucleotide transhydrogenase	1456573_x_at	1,70
Nov	nephroblastoma overexpressed gene	1426852_x_at	0,67
Nov	nephroblastoma overexpressed gene	1426851_a_at	0,70
Npas2	neuronal PAS domain protein 2	1421037_at	—
Npdc1	neural proliferation, differentiation and control gene	1415919_at	0,67
Nrap	nebulin-related anchoring protein	1421253_at	1,97
Nrip1	nuclear receptor interacting protein 1	1449089_at	—
Nt5c3	5'-nucleotidase, cytosolic III	1451050_at	1,78
Nuak1	NUAK family, SNF1-like kinase, 1	1438684_at	0,65
Nup210	nucleoporin 210	1417585_at	3,14
Nupr1	nuclear protein 1	1419665_a_at	0,63
Nupr1	nuclear protein 1	1419666_x_at	0,67
Oasl2	2'-5' oligoadenylate synthetase-like 2	1453196_a_at	0,62
Obscn	obscurin, cytoskeletal calmodulin and titin-interacti	1443632_at	3,56
Odc1	ornithine decarboxylase, structural 1	1438761_a_at	1,42
Ogdh	oxoglutarate dehydrogenase (lipoamide)	1451274_at	1,58
Ogn	osteoglycin	1419662_at	0,56
Ogn	osteoglycin	1419663_at	0,72
Olfml3	olfactomedin-like 3	1448475_at	0,59
Oplah	5-oxoprolinase (ATP-hydrolysing)	1424359_at	2,04
ORF63	open reading frame 63	1451810_at	3,97
Ostc	oligosaccharyltransferase complex subunit	1449139_at	0,67
Pa2g4	proliferation-associated 2G4	1423060_at	1,41
Pabpc1	poly(A) binding protein, cytoplasmic 1	1453840_at	1,93
Pamr1	peptidase domain containing associated with muscle	1433529_at	0,31
Paqr9	progestin and adipoQ receptor family member IX	1455025_at	2,77
Parva	parvin, alpha	1431375_s_at	0,62
Parvb	parvin, beta	1438672_at	1,58
Pbx1	pre B-cell leukemia transcription factor 1	1440037_at	1,91
Pcdh17	protocadherin 17	1453070_at	0,47
Pcm1	pericentriolar material 1	1436908_at	0,68

Pcna	proliferating cell nuclear antigen	1417947_at	1,43
Pcolce	procollagen C-endopeptidase enhancer protein	1448433_a_at	0,59
Pcolce	procollagen C-endopeptidase enhancer protein	1437165_a_at	0,69
Pcsk6	proprotein convertase subtilisin/kexin type 6	1426981_at	0,61
Pcyt1a	phosphate cytidylyltransferase 1, choline, alpha isof	1421957_a_at	1,55
Pdap1	PDGFA associated protein 1	1434020_at	—
Pde4dip	phosphodiesterase 4D interacting protein (myomega	1460426_at	2,41
Pdgfa	platelet derived growth factor, alpha	1418711_at	0,70
Pdgfc	platelet-derived growth factor, C polypeptide	1419123_a_at	0,73
Pdgfd	platelet-derived growth factor, D polypeptide	1426319_at	0,35
Pdgfra	platelet derived growth factor receptor, alpha polype	1421917_at	0,74
Pdlim2	PDZ and LIM domain 2	1423946_at	0,61
Pdlim3	PDZ and LIM domain 3	1443299_at	3,22
Pdpk1	3-phosphoinositide dependent protein kinase 1	1416501_at	1,72
Pdpr	pyruvate dehydrogenase phosphatase regulatory sub	1434480_at	1,81
Pdzk1ip1	PDZK1 interacting protein 1	1417689_a_at	2,93
Pea15a	phosphoprotein enriched in astrocytes 15A	1416407_at	0,72
Per3	period homolog 3 (Drosophila)	1421087_at	—
Pfas	phosphoribosylformylglycinamidine synthase (FGA	1455496_at	1,80
Pfdn4	prefoldin 4	1454888_at	0,76
Phex	phosphate regulating gene with homologies to endop	1421979_at	0,71
Phkb	phosphorylase kinase beta	1434511_at	1,74
Pid1	phosphotyrosine interaction domain containing 1	1436999_at	0,52
Piga	phosphatidylinositol glycan anchor biosynthesis, cla	1427305_at	2,02
Pigq	phosphatidylinositol glycan anchor biosynthesis, cla	1438652_x_at	1,69
Pigq	phosphatidylinositol glycan anchor biosynthesis, cla	1415946_at	2,06
Pik3r1	phosphatidylinositol 3-kinase, regulatory subunit, po	1438682_at	1,48
Pim1	proviral integration site 1	1435458_at	1,86
Pisd-ps3	phosphatidylserine decarboxylase, pseudogene 3	1453145_at	—
Plat	plasminogen activator, tissue	1415806_at	0,57
Pls3	plastin 3 (T-isoform)	1423725_at	0,69
Pnrc2	proline-rich nuclear receptor coactivator 2	1416186_at	0,70
Pofut2	protein O-fucosyltransferase 2	1435056_x_at	0,72
Ppargc1a	peroxisome proliferative activated receptor, gamma,	1460336_at	2,31
Ppbp	pro-platelet basic protein	1418480_at	2,33
Ppfibp2	PTPRF interacting protein, binding protein 2 (liprin	1417801_a_at	0,60
Ppm1l	protein phosphatase 1 (formerly 2C)-like	1438012_at	2,91
Ppox	protoporphyrinogen oxidase	1416618_at	2,25
Ppp1r15a	protein phosphatase 1, regulatory (inhibitor) subunit	1448325_at	2,13
Praf2	PRA1 domain family 2	1416782_s_at	0,57
Prcp	prolylcarboxypeptidase (angiotensinase C)	1452191_at	0,60
Prcp	prolylcarboxypeptidase (angiotensinase C)	1452190_at	0,66
Prdx2	peroxiredoxin 2	1430979_a_at	0,39
Prdx4	peroxiredoxin 4	1416166_a_at	0,66
Prg2	proteoglycan 2, bone marrow	1422873_at	—
Prkaa2	protein kinase, AMP-activated, alpha 2 catalytic sub	1429463_at	2,33
Prkar2b	protein kinase, cAMP dependent regulatory, type II l	1456475_s_at	1,78
Prkar2b	protein kinase, cAMP dependent regulatory, type II l	1438664_at	1,90
Prkcdbp	protein kinase C, delta binding protein	1423771_at	0,62
Prkcq	protein kinase C, theta	1426044_a_at	1,79
Prss35	protease, serine, 35	1434195_at	0,75
Prtn3	proteinase 3	1419669_at	—
Psmd11	proteasome (prosome, macropain) 26S subunit, non-	1437080_s_at	1,31
Ptch1	patched homolog 1	1428853_at	—
Ptgis	prostaglandin I2 (prostacyclin) synthase	1448816_at	0,68
Pth1r	parathyroid hormone 1 receptor	1417092_at	0,60

Ptp4a3	protein tyrosine phosphatase 4a3	1418181_at	1,57
Pttg1ip	pituitary tumor-transforming 1 interacting protein	1437342_x_at	0,77
Pum1	pumilio 1 (Drosophila)	1456054_a_at	—
Pycr1	pyrroline-5-carboxylate reductase 1	1424556_at	0,60
Rad21	RAD21 homolog (S. pombe)	1416161_at	1,55
Rasal2	RAS protein activator like 2	1444671_at	—
Rb1	retinoblastoma 1	1417850_at	1,64
Rbfox1	RNA binding protein, fox-1 homolog (C. elegans) 1	1418314_a_at	2,15
Rbm25	RNA binding motif protein 25	1437862_at	—
Rbm3	RNA binding motif protein 3	1429169_at	1,97
Rbm38	RNA binding motif protein 38	1421265_a_at	2,55
Rbm39	RNA binding motif protein 39	1442745_x_at	—
Rbms3	RNA binding motif, single stranded interacting prote	1436938_at	0,69
Rc3h2	Ring finger and CCCH-type zinc finger domains 2	1460086_at	1,85
Rcbtb2	regulator of chromosome condensation (RCC1) and	1416390_at	0,67
Rcn1	reticulocalbin 1	1417090_at	0,61
Rcn3	reticulocalbin 3, EF-hand calcium binding domain	1424382_at	0,60
Rerg	RAS-like, estrogen-regulated, growth-inhibitor	1451236_at	0,56
Rhag	Rhesus blood group-associated A glycoprotein	1419014_at	4,69
Rhd	Rh blood group, D antigen	1417049_at	4,11
Rif1	Rap1 interacting factor 1 homolog (yeast)	1442939_at	1,92
Rin2	Ras and Rab interactor 2	1426368_at	0,58
Rnase4	ribonuclease, RNase A family 4	1422603_at	0,56
Rnd3	Rho family GTPase 3	1416700_at	0,61
Robo1	roundabout homolog 1 (Drosophila)	1427231_at	0,54
Rpia	ribose 5-phosphate isomerase A	1418337_at	1,70
Rpl39l	ribosomal protein L39-like	1423327_at	0,64
Rps3	ribosomal protein S3	1455600_at	0,44
Rps6ka1	ribosomal protein S6 kinase polypeptide 1	1416896_at	2,00
Rrm2	ribonucleotide reductase M2	1448226_at	2,00
Rrm2	ribonucleotide reductase M2	1434437_x_at	2,02
Rsad2	radical S-adenosyl methionine domain containing 2	1421008_at	2,42
Rsad2	radical S-adenosyl methionine domain containing 2	1436058_at	2,44
Rsad2	radical S-adenosyl methionine domain containing 2	1421009_at	2,95
Rsf1	remodeling and spacing factor 1	1457751_at	0,51
Rtp4	receptor transporter protein 4	1418580_at	0,64
Ryr1	ryanodine receptor 1, skeletal muscle	1427306_at	2,68
Ryr1	ryanodine receptor 1, skeletal muscle	1457347_at	6,73
S100a10	S100 calcium binding protein A10 (calpactin)	1416762_at	0,64
S100a16	S100 calcium binding protein A16	1447676_x_at	0,72
S100a16	S100 calcium binding protein A16	1425560_a_at	0,74
S1pr3	sphingosine-1-phosphate receptor 3	1437173_at	0,68
Satb1	special AT-rich sequence binding protein 1	1416007_at	1,54
Scara3	scavenger receptor class A, member 3	1427020_at	0,69
Sdc2	syndecan 2	1417012_at	0,56
Sec22b	SEC22 vesicle trafficking protein homolog B (S. cer	1449063_at	0,69
Sec23a	SEC23A (S. cerevisiae)	1423347_at	0,75
Sec31a	Sec31 homolog A (S. cerevisiae)	1453014_a_at	0,69
Sec61a1	Sec61 alpha 1 subunit (S. cerevisiae)	1448242_at	0,61
Sec61a1	Sec61 alpha 1 subunit (S. cerevisiae)	1416189_a_at	0,68
Selk	selenoprotein K	1447909_s_at	0,75
Selm	selenoprotein M	1424394_at	0,58
Sema3c	sema domain, immunoglobulin domain (Ig), short ba	1429348_at	0,58
Serf1	small EDRK-rich factor 1	1434291_a_at	0,64
Serpinh1	serine (or cysteine) peptidase inhibitor, clade H, mer	1456733_x_at	0,38
Serpinh1	serine (or cysteine) peptidase inhibitor, clade H, mer	1450843_a_at	0,65

Setd8	SET domain containing (lysine methyltransferase) 8	1426406_at	1,79
Sf3b2	splicing factor 3b, subunit 2	1429362_a_at	1,53
Sfrp4	secreted frizzled-related protein 4	1451031_at	0,67
Sgol1	shugoshin-like 1 (S. pombe)	1418919_at	1,80
Sh3bgrl	SH3-binding domain glutamic acid-rich protein like	1421871_at	0,66
Sh3pxd2b	SH3 and PX domains 2B	1435644_at	0,55
Sin3b	transcriptional regulator, SIN3B (yeast)	1455039_a_at	0,70
Slbp	stem-loop binding protein	1460168_at	1,67
Slc13a5	solute carrier family 13 (sodium-dependent citrate tr	1435936_at	0,58
Slc14a1	solute carrier family 14 (urea transporter), member 1	1428114_at	3,91
Slc16a1	solute carrier family 16 (monocarboxylic acid transp	1415802_at	1,84
Slc16a4	solute carrier family 16 (monocarboxylic acid transp	1426082_a_at	0,50
Slc20a1	solute carrier family 20, member 1	1448568_a_at	1,63
Slc25a37	solute carrier family 25, member 37	1417750_a_at	3,42
Slc2a13	solute carrier family 2 (facilitated glucose transporte	1435387_at	0,55
Slc2a4	solute carrier family 2 (facilitated glucose transporte	1415959_at	3,51
Slc38a5	solute carrier family 38, member 5	1454622_at	2,57
Slc43a1	solute carrier family 43, member 1	1453255_at	2,02
Slc43a3	solute carrier family 43, member 3	1422788_at	1,53
Slc4a1	solute carrier family 4 (anion exchanger), member 1	1434502_x_at	1,91
Slc4a1	solute carrier family 4 (anion exchanger), member 1	1416464_at	4,32
Slc6a20a	solute carrier family 6 (neurotransmitter transporter)	1427221_at	5,04
Slc7a5	solute carrier family 7 (cationic amino acid transpor	1418326_at	1,79
Sln	sarcolipin	1420884_at	1,97
Smc2	structural maintenance of chromosomes 2	1448635_at	1,54
Smc2	structural maintenance of chromosomes 2	1429660_s_at	2,03
Smc4	structural maintenance of chromosomes 4	1427275_at	1,82
Smc4	structural maintenance of chromosomes 4	1427276_at	1,91
Smo	smoothened homolog (Drosophila)	1427048_at	0,59
Smo	smoothened homolog (Drosophila)	1427049_s_at	0,63
Smpd3	sphingomyelin phosphodiesterase 3, neutral	1422779_at	0,52
Smpd3	sphingomyelin phosphodiesterase 3, neutral	1438665_at	0,59
Smtnl2	smoothelin-like 2	1455794_at	2,82
Snap47	synaptosomal-associated protein, 47	1423745_at	0,71
Snap47	synaptosomal-associated protein, 47	1438176_x_at	0,77
Snca	synuclein, alpha	1436853_a_at	3,03
Snca	synuclein, alpha	1418493_a_at	3,54
Snhg6	small nucleolar RNA host gene (non-protein coding)	1436506_a_at	0,53
Snord123	small nucleolar RNA, C/D box 123	1429027_at	0,66
Sorl1	sortilin-related receptor, LDLR class A repeats-conta	1453003_at	2,27
Sorl1	sortilin-related receptor, LDLR class A repeats-conta	1426258_at	2,41
Sox11	SRY-box containing gene 11	1453125_at	1,94
Sox4	SRY-box containing gene 4	1419155_a_at	0,62
Sox4	SRY-box containing gene 4	1419156_at	0,63
Sox4	SRY-box containing gene 4	1433575_at	0,70
Sox6	SRY-box containing gene 6	1434918_at	1,74
Sox6	SRY-box containing gene 6	1447655_x_at	1,89
Sox6	SRY-box containing gene 6	1427677_a_at	2,02
Sp1	trans-acting transcription factor 1	1418180_at	1,59
Sp7	Sp7 transcription factor 7	1418425_at	0,61
Spire1	spire homolog 1 (Drosophila)	1455409_at	1,66
Spna1	spectrin alpha 1	1421277_at	4,45
Spna1	spectrin alpha 1	1421278_s_at	4,67
Spna2	spectrin alpha 2	1427889_at	0,74
Spnb2	spectrin beta 2	1444089_at	0,62
Spon1	spondin 1, (f-spondin) extracellular matrix protein	1451342_at	0,58

Spred1	sprouty protein with EVH-1 domain 1, related seque	1460116_s_at	0,67
Srp54a /// Srp54b /// S	signal recognition particle 54A /// signal recognition	1425665_a_at	0,68
Srrm2	serine/arginine repetitive matrix 2	1438688_at	—
Srsf4	serine/arginine-rich splicing factor 4	1448778_at	1,43
Ssr2	signal sequence receptor, beta	1449930_a_at	0,63
St3gal5	ST3 beta-galactoside alpha-2,3-sialyltransferase 5	1460241_a_at	1,98
Stom	stomatin	1438910_a_at	1,81
Stradb	STE20-related kinase adaptor beta	1433544_at	1,97
Stt3a	STT3, subunit of the oligosaccharyltransferase comp	1455824_x_at	0,71
Sulf1	sulfatase 1	1438200_at	0,52
Sulf1	sulfatase 1	1436319_at	0,57
Sumf1	sulfatase modifying factor 1	1424603_at	0,56
Sumf1	sulfatase modifying factor 1	1424604_s_at	0,75
Surf4	surfeit gene 4	1436797_a_at	0,66
Surf4	surfeit gene 4	1416213_x_at	0,67
Surf4	surfeit gene 4	1448255_a_at	0,67
Surf4	surfeit gene 4	1455822_x_at	0,74
Suz12	suppressor of zeste 12 homolog (Drosophila)	1420021_s_at	1,49
Suz12	suppressor of zeste 12 homolog (Drosophila)	1427253_s_at	1,64
Suz12	suppressor of zeste 12 homolog (Drosophila)	1452364_at	1,66
Synm	synemin, intermediate filament protein	1457275_at	1,84
Sypl	synaptophysin-like protein	1435082_at	1,59
Tab3	TGF-beta activated kinase 1/MAP3K7 binding prote	1428762_at	1,75
Taf1d	TATA box binding protein (Tbp)-associated factor, R	1457292_at	1,79
Tagln	transgelin	1423505_at	0,47
Tal1	T-cell acute lymphocytic leukemia 1	1449389_at	3,20
Tbca	tubulin cofactor A	1437907_a_at	0,77
Tbcel	tubulin folding cofactor E-like	1454930_at	1,58
Tceal7	transcription elongation factor A (SII)-like 7	1435585_at	—
Tceb2	transcription elongation factor B (SIII), polypeptide	1436949_a_at	0,77
Tcf7	transcription factor 7, T-cell specific	1433471_at	0,54
Tex2	testis expressed gene 2	1452213_at	1,81
Tfdp2	transcription factor Dp 2	1437174_at	2,26
Tfdp2	transcription factor Dp 2	1443962_at	2,58
Tfpi2	tissue factor pathway inhibitor 2	1418547_at	0,64
Tgfb2	transforming growth factor, beta 2	1450923_at	0,56
Tgs1	trimethylguanosine synthase homolog (S. cerevisiae	1450400_at	1,88
Thbs2	thrombospondin 2	1447862_x_at	0,58
Thbs2	thrombospondin 2	1450663_at	0,58
Thbs2	thrombospondin 2	1422571_at	0,60
Thrsp	thyroid hormone responsive SPOT14 homolog (Ratt	1424737_at	—
Thy1	thymus cell antigen 1, theta	1423135_at	0,56
Timp2	tissue inhibitor of metalloproteinase 2	1454677_at	0,68
Timp2	tissue inhibitor of metalloproteinase 2	1450040_at	0,70
Tm2d2	TM2 domain containing 2	1456663_x_at	0,57
Tmc7	transmembrane channel-like gene family 7	1456981_at	0,39
Tmcc2	transmembrane and coiled-coil domains 2	1428108_x_at	3,22
Tmcc2	transmembrane and coiled-coil domains 2	1452666_a_at	3,54
Tmem119	transmembrane protein 119	1451344_at	0,47
Tmem159	transmembrane protein 159	1426436_at	0,55
Tmem167	transmembrane protein 167	1425780_a_at	0,71
Tmem176a	transmembrane protein 176A	1441811_x_at	0,64
Tmem176b	transmembrane protein 176B	1418004_a_at	0,75
Tmem56	transmembrane protein 56	1434553_at	2,37
Tmsb10	thymosin, beta 10	1417219_s_at	0,64
Tmsb10	thymosin, beta 10	1436902_x_at	0,71

Tnc	tenascin C	1416342_at	0,66
Tnmd	tenomodulin	1417979_at	0,72
Tnn	tenascin N	1442140_at	0,69
Tpm4	tropomyosin 4	1433883_at	0,71
Tppp3	tubulin polymerization-promoting protein family me	1416713_at	0,56
Trak2	trafficking protein, kinesin binding 2	1435016_at	1,66
Tram2	translocating chain-associating membrane protein 2	1439539_at	0,66
Trappc10	trafficking protein particle complex 10	1435430_at	1,59
Trim10	tripartite motif-containing 10	1419311_at	3,33
Trim12a	tripartite motif-containing 12A	1437432_a_at	0,23
Trim30a	tripartite motif-containing 30A	1417961_a_at	0,59
Trim30d	tripartite motif-containing 30D	1435665_at	0,33
Trim34	tripartite motif-containing 34	1424857_a_at	0,31
Trim54	tripartite motif-containing 54	1419440_at	2,87
Trim59	tripartite motif-containing 59	1416118_at	1,82
Trim7	tripartite motif-containing 7	1421398_at	7,96
Tspan13	tetraspanin 13	1460239_at	1,59
Tspan31	tetraspanin 31	1416556_at	0,70
Tspan31	tetraspanin 31	1430029_a_at	0,71
Tspan33	tetraspanin 33	1425157_x_at	2,68
Tspan33	tetraspanin 33	1451608_a_at	4,34
Tspan33	tetraspanin 33	1455618_x_at	4,91
Tspan6	tetraspanin 6	1416872_at	0,59
Tspan6	tetraspanin 6	1448501_at	0,60
Tspan8	tetraspanin 8	1424649_a_at	2,50
Ttn	titin	1444638_at	3,48
Ttn	titin	1444083_at	3,72
Tuba4a	tubulin, alpha 4A	1417374_at	2,18
Tubb1	tubulin, beta 1	1444214_at	2,99
Txndc17	thioredoxin domain containing 17	1423035_s_at	0,74
Ubac1	ubiquitin associated domain containing 1	1416963_at	1,84
Ube2c	ubiquitin-conjugating enzyme E2C	1452954_at	2,49
Ube2d1	ubiquitin-conjugating enzyme E2D 1, UBC4/5 hom	1424062_at	—
Ube2i	ubiquitin-conjugating enzyme E2I	1453189_at	1,73
Ube2o	ubiquitin-conjugating enzyme E2O	1433765_at	2,19
Ufm1	ubiquitin-fold modifier 1	1449263_at	0,76
Uhrf1	ubiquitin-like, containing PHD and RING finger do	1415810_at	1,80
Urod	uroporphyrinogen decarboxylase	1443849_x_at	1,57
Use1	unconventional SNARE in the ER 1 homolog (S. ce	1451130_at	0,62
Use1	unconventional SNARE in the ER 1 homolog (S. ce	1423817_s_at	0,65
Usp32	ubiquitin specific peptidase 32	1436159_at	1,58
Usp32	ubiquitin specific peptidase 32	1459857_at	2,95
Vasn	vasorin	1455812_x_at	0,63
Vasn	vasorin	1427894_at	0,64
Vcam1	vascular cell adhesion molecule 1	1448162_at	1,49
Vcan	versican	1427256_at	0,62
Vgll2	vestigial like 2 homolog (Drosophila)	1436361_at	2,27
Vps37a	vacuolar protein sorting 37A (yeast)	1429363_at	1,91
Vwa5a	von Willebrand factor A domain containing 5A	1426221_at	0,35
Wdfy1	WD repeat and FYVE domain containing 1	1424749_at	2,79
Wdfy1	WD repeat and FYVE domain containing 1	1435588_at	2,82
Wdfy1	WD repeat and FYVE domain containing 1	1437358_at	3,09
Wdr1	WD repeat domain 1	1423054_at	0,70
Wdr26	WD repeat domain 26	1438234_at	—
Wif1	Wnt inhibitory factor 1	1425425_a_at	0,72
Wisp1	WNT1 inducible signaling pathway protein 1	1448594_at	0,62

Wnk1	WNK lysine deficient protein kinase 1	1436746_at	1,87
Xist	inactive X specific transcripts	1427262_at	—
Xpo7	exportin 7	1415682_at	2,11
Xpo7	exportin 7	1439411_a_at	2,12
Ydjc	YdjC homolog (bacterial)	1428550_at	0,61
Ypel4	yippee-like 4 (Drosophila)	1434501_at	3,54
Zbtb16	zinc finger and BTB domain containing 16	1439163_at	2,36
Zc3h11a	zinc finger CCCH type containing 11A	1426360_at	—
Zdhhc14	zinc finger, DHHC domain containing 14	1437614_x_at	1,50
Zdhhc14	zinc finger, DHHC domain containing 14	1438619_x_at	1,56
Zeb2	zinc finger E-box binding homeobox 2	1454200_at	—
Zfhx4	zinc finger homeodomain 4	1437556_at	0,61
Zfhx4	zinc finger homeodomain 4	1421433_at	0,65
Zfp110	zinc finger protein 110	1437236_a_at	0,72
Zfp260	zinc finger protein 260	1419165_at	0,67
Zfp354c	zinc finger protein 354C	1439044_at	0,67
1110020G09Rik	RIKEN cDNA 1110020G09 gene	1437287_at	1,85
1110036O03Rik	RIKEN cDNA 1110036O03 gene	1455288_at	0,61
1110049F12Rik	RIKEN cDNA 1110049F12 gene	1428675_at	1,41
1300017J02Rik	RIKEN cDNA 1300017J02 gene	1424722_at	7,54
1600012H06Rik	RIKEN cDNA 1600012H06 gene	1428217_at	0,65
1700020C11Rik	RIKEN cDNA 1700020C11 gene	1424223_at	1,95
1700066M21Rik	RIKEN cDNA 1700066M21 gene	1434422_at	0,67
2010309G21Rik /// Ig	RIKEN cDNA 2010309G21 gene /// immunoglobuli	1428719_at	2,52
2010309G21Rik /// Ig	RIKEN cDNA 2010309G21 gene /// immunoglobuli	1428720_s_at	2,46
2610507B11Rik	RIKEN cDNA 2610507B11 gene	1455905_at	—
2810021G02Rik	RIKEN cDNA 2810021G02 gene	1427349_x_at	0,73
2810453I06Rik	RIKEN cDNA 2810453I06 gene	1418389_at	1,96
2810453I06Rik	RIKEN cDNA 2810453I06 gene	1451975_at	2,16
4432414F05Rik	RIKEN cDNA 4432414F05 gene	1453976_at	1,97
4930422I07Rik	RIKEN cDNA 4930422I07 gene	1458491_at	2,73
4930430F08Rik	RIKEN cDNA 4930430F08 gene	1457754_at	8,32
4933411D12Rik	RIKEN cDNA 4933411D12 gene	1442843_at	1,70
5430401H09Rik	RIKEN cDNA 5430401H09 gene	1454193_at	3,60
5430435G22Rik	RIKEN cDNA 5430435G22 gene	1424987_at	0,67
5730469M10Rik	RIKEN cDNA 5730469M10 gene	1447774_x_at	2,82
5730469M10Rik	RIKEN cDNA 5730469M10 gene	1452716_at	3,13
5730508B09Rik	RIKEN cDNA 5730508B09 gene	1429678_at	1,82
5730508B09Rik	RIKEN cDNA 5730508B09 gene	1447100_s_at	—
5830474E16Rik	RIKEN cDNA 5830474E16 gene	1433110_at	2,68
6030458C11Rik	RIKEN cDNA 6030458C11 gene	1433860_at	1,39
6330564D18Rik	RIKEN cDNA 6330564D18 gene	1460624_at	1,79
9030425E11Rik	RIKEN cDNA 9030425E11 gene	1448251_at	0,51
9130009I01Rik	RIKEN cDNA 9130009I01 gene	1430498_at	3,25
9130011J15Rik	RIKEN cDNA 9130011J15 gene	1426646_at	0,67
9130011J15Rik	RIKEN cDNA 9130011J15 gene	1415705_at	0,70
9930013L23Rik	RIKEN cDNA 9930013L23 gene	1429987_at	0,54
A130071D04Rik	RIKEN cDNA A130071D04 gene	1438052_at	2,20
A230046K03Rik	RIKEN cDNA A230046K03 gene	1439450_x_at	—

-Krm2	Lrp5-/-			
SD	FC	SD		
1,29	—	—		
1,09	—	—		
0,31	—	—		
1,90	—	—		
1,06	—	—		
1,29	—	—		
0,87	—	—		
1,31	—	—		
0,13	—	—		
0,23	—	—		
0,66	—	—		
0,08	0,66	0,13		
0,07	—	—		
0,11	—	—		
2,80	—	—		
—	1,83	0,21		
0,37	—	—		
1,66	—	—		
1,42	—	—		
0,31	—	—		
0,24	—	—		
0,94	—	—		
—	1,49	0,37		
2,84	—	—		
1,98	—	—		
0,27	—	—		
1,50	—	—		
0,98	—	—		
—	0,61	0,18		
0,21	—	—		
0,36	—	—		
0,98	—	—		
0,24	0,66	0,23		
0,21	—	—		
0,13	—	—		
0,17	—	—		
0,42	—	—		
0,08	—	—		
0,84	—	—		
—	1,59	0,36		
3,54	—	—		
1,10	—	—		
41,18	—	—		
0,27	—	—		
0,17	—	—		
0,13	—	—		
0,15	—	—		
1,31	—	—		
0,13	—	—		
0,12	—	—		
0,19	—	—		

0,03	0,70	0,09		
2,55	—	—		
0,55	—	—		
0,11	—	—		
0,15	—	—		
0,15	—	—		
0,80	—	—		
1,37	—	—		
0,56	—	—		
—	1,41	0,17		
0,16	—	—		
0,17	—	—		
0,37	—	—		
0,14	—	—		
2,04	—	—		
0,14	—	—		
0,20	—	—		
0,63	—	—		
0,80	2,09	0,79		
0,53	—	—		
0,15	—	—		
0,34	—	—		
0,15	—	—		
0,23	—	—		
0,38	—	—		
—	3,00	1,24		
0,07	—	—		
0,26	—	—		
—	0,61	0,08		
1,81	—	—		
1,70	—	—		
0,27	—	—		
0,16	—	—		
0,14	—	—		
0,55	—	—		
0,04	—	—		
0,13	—	—		
0,84	1,80	0,59		
0,97	—	—		
1,34	1,77	0,65		
0,31	—	—		
1,17	—	—		
0,19	—	—		
0,27	—	—		
1,35	—	—		
1,65	—	—		
0,23	—	—		
0,22	—	—		
0,25	0,66	0,11		
2,58	—	—		
1,44	—	—		
0,15	—	—		
0,08	—	—		
0,11	—	—		
0,35	—	—		
0,52	—	—		

0,23	—	—		
0,61	—	—		
0,32	—	—		
0,27	—	—		
0,63	—	—		
0,62	—	—		
1,32	—	—		
0,19	—	—		
0,09	—	—		
0,87	—	—		
0,58	—	—		
0,10	—	—		
0,14	—	—		
1,62	—	—		
0,53	—	—		
0,48	—	—		
0,17	—	—		
0,23	—	—		
0,11	—	—		
0,45	—	—		
0,37	—	—		
0,42	—	—		
1,36	—	—		
10,84	—	—		
0,21	—	—		
0,20	—	—		
0,16	—	—		
0,72	—	—		
—	1,65	0,31		
0,26	—	—		
0,74	—	—		
0,85	—	—		
2,34	—	—		
0,35	2,00	0,53		
0,09	—	—		
0,24	—	—		
0,89	—	—		
0,16	—	—		
0,19	—	—		
0,19	—	—		
—	0,77	0,23		
0,29	—	—		
0,28	—	—		
0,29	—	—		
0,86	—	—		
0,27	—	—		
0,28	—	—		
0,17	—	—		
0,24	—	—		
0,14	—	—		
0,15	—	—		
0,07	—	—		
1,23	—	—		
0,43	—	—		
0,28	—	—		
0,80	—	—		

0,84	—	—		
—	0,75	0,09		
0,20	—	—		
0,16	—	—		
—	1,53	0,26		
0,18	—	—		
0,19	—	—		
—	0,68	0,29		
—	1,64	0,32		
—	1,51	0,26		
1,33	—	—		
0,16	—	—		
0,13	—	—		
0,37	—	—		
0,18	1,62	0,27		
0,30	—	—		
2,38	—	—		
0,11	—	—		
0,88	—	—		
0,16	—	—		
0,18	—	—		
0,76	1,96	0,86		
0,22	—	—		
0,16	—	—		
1,37	—	—		
0,27	—	—		
—	1,75	0,29		
—	2,01	0,49		
0,61	—	—		
0,13	—	—		
0,26	—	—		
0,39	—	—		
0,29	1,44	0,19		
1,39	—	—		
0,30	—	—		
0,37	—	—		
0,67	—	—		
0,44	—	—		
0,11	—	—		
0,40	—	—		
0,03	—	—		
0,18	—	—		
0,22	—	—		
0,25	—	—		
0,12	—	—		
1,81	—	—		
1,59	—	—		
1,65	—	—		
0,31	—	—		
1,08	—	—		
0,07	—	—		
0,07	—	—		
0,32	—	—		
0,17	—	—		
0,81	—	—		
0,49	—	—		

0,22	—	—		
0,69	—	—		
0,28	1,82	0,39		
0,89	2,10	0,56		
0,11	—	—		
1,50	—	—		
0,17	1,45	0,18		
0,09	—	—		
0,20	—	—		
0,45	—	—		
0,03	—	—		
0,08	—	—		
0,11	—	—		
1,35	—	—		
0,21	—	—		
4,05	3,63	4,01		
3,17	—	—		
0,76	—	—		
0,68	—	—		
0,45	—	—		
0,45	—	—		
0,25	—	—		
0,21	—	—		
1,23	—	—		
0,22	—	—		
2,69	—	—		
0,12	—	—		
0,22	—	—		
0,06	—	—		
0,20	—	—		
1,40	—	—		
0,60	—	—		
0,23	—	—		
0,17	—	—		
0,29	—	—		
0,08	—	—		
0,23	0,69	0,16		
—	0,73	0,15		
1,06	—	—		
0,54	—	—		
0,30	—	—		
0,36	—	—		
2,73	—	—		
0,44	—	—		
0,69	—	—		
0,44	—	—		
0,55	—	—		
0,70	—	—		
0,26	—	—		
0,95	—	—		
1,35	—	—		
0,61	—	—		
0,26	—	—		
0,23	0,72	0,18		
0,27	—	—		
0,28	—	—		

—	0,80	0,31		
0,86	—	—		
0,30	0,78	0,20		
0,32	—	—		
0,27	—	—		
—	1,60	0,45		
0,82	—	—		
0,55	—	—		
0,53	—	—		
1,39	—	—		
0,34	—	—		
—	1,67	0,45		
—	1,94	0,38		
0,74	—	—		
1,27	—	—		
0,79	—	—		
1,13	—	—		
0,67	—	—		
0,59	—	—		
2,62	—	—		
0,18	—	—		
0,26	—	—		
0,27	—	—		
0,09	—	—		
0,09	—	—		
0,19	—	—		
0,03	—	—		
0,74	—	—		
0,54	—	—		
0,13	—	—		
1,39	—	—		
0,63	1,97	0,54		
0,57	2,03	0,54		
0,15	—	—		
1,27	—	—		
0,46	—	—		
0,70	1,84	0,73		
0,16	—	—		
0,13	—	—		
0,15	—	—		
0,37	—	—		
1,75	—	—		
0,54	1,49	0,42		
0,22	0,67	0,17		
0,29	—	—		
0,27	—	—		
0,50	—	—		
0,04	—	—		
0,27	—	—		
0,28	—	—		
0,20	—	—		
0,34	—	—		
0,70	—	—		
0,16	—	—		
0,23	—	—		
2,62	—	—		

3,34	3,25	3,35		
0,69	—	—		
1,08	—	—		
0,13	—	—		
0,41	—	—		
0,25	—	—		
34,92	—	—		
0,96	—	—		
35,84	—	—		
0,26	—	—		
0,48	—	—		
0,57	—	—		
0,16	—	—		
0,39	—	—		
2,41	2,86	2,78		
0,20	1,32	0,14		
0,68	—	—		
1,33	—	—		
0,23	—	—		
0,64	—	—		
0,15	—	—		
0,15	—	—		
1,43	—	—		
0,29	—	—		
0,29	—	—		
0,33	—	—		
2,96	—	—		
0,29	—	—		
0,20	0,70	0,30		
0,39	—	—		
0,52	—	—		
0,20	—	—		
0,07	—	—		
0,13	—	—		
0,07	—	—		
1,32	1,73	0,94		
1,50	1,67	0,96		
1,32	1,88	1,05		
2,00	—	—		
2,33	—	—		
3,26	—	—		
10,69	—	—		
12,22	—	—		
1,59	—	—		
6,59	—	—		
1,25	—	—		
3,08	—	—		
3,19	—	—		
0,22	—	—		
0,17	—	—		
1,25	—	—		
0,10	—	—		
0,71	—	—		
0,61	—	—		
0,88	—	—		
0,24	—	—		

0,36	—	—		
0,74	—	—		
0,16	—	—		
—	2,81	1,38		
0,15	—	—		
0,26	0,70	0,21		
—	1,36	0,24		
—	1,49	0,33		
—	1,60	0,21		
0,08	—	—		
0,16	—	—		
0,16	—	—		
0,31	—	—		
0,27	—	—		
0,27	—	—		
0,32	0,78	0,24		
0,38	—	—		
4,10	—	—		
0,36	—	—		
1,22	—	—		
0,64	—	—		
3,63	—	—		
3,62	—	—		
3,35	—	—		
0,15	—	—		
0,09	—	—		
0,13	—	—		
0,48	—	—		
1,11	—	—		
0,23	—	—		
0,17	—	—		
—	0,76	0,11		
0,58	—	—		
0,91	—	—		
0,07	—	—		
0,08	—	—		
—	1,49	0,33		
0,13	—	—		
0,63	2,14	0,70		
0,10	—	—		
0,22	—	—		
0,11	—	—		
—	0,72	0,08		
—	1,33	0,10		
0,24	—	—		
0,25	—	—		
0,12	—	—		
0,25	—	—		
0,25	—	—		
0,15	—	—		
0,24	—	—		
0,07	0,69	0,05		
0,18	—	—		
—	1,35	0,28		
0,10	—	—		
0,08	—	—		

0,85	—	—		
—	1,44	0,04		
0,29	—	—		
11,32	—	—		
0,07	—	—		
0,65	—	—		
0,51	—	—		
0,28	—	—		
0,05	—	—		
0,26	—	—		
1,11	—	—		
0,07	—	—		
0,07	—	—		
0,06	—	—		
0,18	—	—		
0,25	—	—		
0,20	0,73	0,16		
0,43	—	—		
0,71	—	—		
0,15	—	—		
0,46	—	—		
0,63	—	—		
0,43	—	—		
0,27	—	—		
0,08	—	—		
0,07	—	—		
0,09	—	—		
0,18	—	—		
0,18	—	—		
0,14	—	—		
0,16	—	—		
0,07	0,57	0,13		
0,08	0,62	0,14		
0,57	—	—		
0,71	—	—		
0,20	—	—		
0,20	—	—		
0,54	—	—		
0,52	—	—		
0,56	—	—		
0,68	—	—		
0,79	—	—		
0,91	—	—		
0,96	—	—		
0,21	—	—		
0,25	—	—		
0,24	—	—		
0,90	—	—		
1,43	—	—		
0,17	—	—		
0,20	—	—		
0,12	—	—		
0,92	1,84	0,94		
—	0,70	0,18		
0,13	—	—		
0,19	—	—		

2,27	—	—		
2,59	—	—		
3,62	—	—		
2,45	3,32	2,61		
0,17	—	—		
0,20	—	—		
0,29	—	—		
0,25	—	—		
0,22	—	—		
0,23	—	—		
1,82	—	—		
0,31	—	—		
0,20	—	—		
0,43	—	—		
0,45	—	—		
0,32	—	—		
0,20	—	—		
0,22	—	—		
0,54	—	—		
0,23	—	—		
0,22	—	—		
0,22	—	—		
0,09	—	—		
0,34	—	—		
0,13	—	—		
0,41	—	—		
0,15	—	—		
0,13	—	—		
—	0,46	0,15		
0,18	—	—		
1,16	—	—		
—	1,93	0,80		
0,59	—	—		
0,25	—	—		
1,96	—	—		
0,21	—	—		
0,21	0,67	0,25		
0,21	—	—		
2,60	—	—		
0,23	—	—		
0,40	—	—		
0,20	—	—		
0,25	—	—		
0,23	—	—		
0,45	—	—		
1,95	—	—		
0,23	—	—		
0,16	—	—		
0,59	—	—		
0,15	0,62	0,32		
1,20	—	—		
0,15	—	—		
0,38	—	—		
0,59	—	—		
0,12	—	—		
0,04	—	—		

0,25	—	—		
0,22	—	—		
0,22	—	—		
0,17	—	—		
0,31	1,38	0,14		
—	1,53	0,20		
1,01	—	—		
0,23	—	—		
0,35	—	—		
0,20	—	—		
0,11	—	—		
0,07	—	—		
2,28	—	—		
0,24	1,47	0,28		
0,48	1,51	0,42		
1,65	—	—		
0,06	—	—		
—	2,04	0,64		
0,35	—	—		
0,18	—	—		
0,24	—	—		
0,63	—	—		
0,09	—	—		
0,51	—	—		
0,40	—	—		
0,73	—	—		
0,17	—	—		
0,61	—	—		
—	1,84	0,58		
0,11	0,64	0,09		
0,24	—	—		
0,08	—	—		
0,28	—	—		
1,22	—	—		
1,57	—	—		
0,12	—	—		
1,78	—	—		
0,72	—	—		
0,93	—	—		
0,31	—	—		
0,11	—	—		
0,08	—	—		
0,17	—	—		
0,26	—	—		
—	0,51	0,20		
1,57	2,11	1,42		
0,63	—	—		
0,72	—	—		
0,16	0,70	0,12		
0,67	—	—		
0,44	—	—		
—	2,08	1,86		
0,21	—	—		
—	0,56	0,28		
0,27	—	—		
0,39	0,66	0,33		

0,47	—	—		
0,11	—	—		
—	1,59	0,33		
0,27	—	—		
0,28	—	—		
—	2,86	0,97		
0,33	—	—		
1,27	—	—		
—	1,55	0,33		
1,26	—	—		
1,57	—	—		
—	1,54	0,40		
0,26	—	—		
0,46	—	—		
0,07	—	—		
0,18	—	—		
0,25	—	—		
0,19	—	—		
2,98	—	—		
2,53	—	—		
0,20	—	—		
0,09	—	—		
0,11	—	—		
0,21	—	—		
0,33	—	—		
0,51	—	—		
0,32	—	—		
0,07	—	—		
0,88	—	—		
0,75	—	—		
0,65	—	—		
1,13	—	—		
0,74	—	—		
1,16	2,25	1,36		
0,17	—	—		
0,23	—	—		
1,46	—	—		
4,49	3,91	2,33		
0,19	—	—		
0,09	0,73	0,08		
0,08	—	—		
0,09	—	—		
0,20	—	—		
0,23	—	—		
0,27	—	—		
0,22	—	—		
0,18	—	—		
0,16	—	—		
0,22	—	—		
0,23	—	—		
0,08	—	—		
0,27	—	—		
0,10	0,58	0,19		
0,17	—	—		
0,21	—	—		
0,20	—	—		

0,43	1,51	0,28		
0,30	1,52	0,12		
0,14	—	—		
0,61	—	—		
0,09	—	—		
0,24	—	—		
0,11	—	—		
0,42	—	—		
0,25	0,61	0,22		
2,54	—	—		
0,52	—	—		
0,20	—	—		
0,35	—	—		
1,65	—	—		
0,24	—	—		
3,48	—	—		
1,24	—	—		
0,93	—	—		
0,30	—	—		
0,57	—	—		
2,78	—	—		
3,56	—	—		
0,36	—	—		
0,81	—	—		
0,42	—	—		
0,26	—	—		
0,52	—	—		
0,37	1,76	0,58		
0,10	—	—		
0,20	—	—		
0,18	—	—		
0,33	—	—		
1,72	—	—		
0,12	—	—		
0,08	—	—		
1,26	—	—		
1,60	—	—		
0,17	—	—		
0,11	—	—		
1,26	—	—		
1,11	—	—		
0,74	—	—		
0,17	—	—		
0,23	—	—		
0,21	—	—		
0,32	—	—		
0,41	—	—		
0,47	1,67	0,47		
0,22	—	—		
0,20	—	—		
0,39	—	—		
2,84	—	—		
2,76	—	—		
0,14	—	—		
0,18	—	—		
0,44	0,73	0,34		

0,08	—	—		
0,17	—	—		
—	1,41	0,23		
0,20	—	—		
0,21	—	—		
0,53	—	—		
0,51	—	—		
0,80	—	—		
0,24	—	—		
0,27	—	—		
0,38	0,72	0,31		
0,15	—	—		
0,16	—	—		
0,17	—	—		
0,16	—	—		
0,24	—	—		
0,15	—	—		
0,27	—	—		
0,36	—	—		
0,35	—	—		
0,67	1,71	0,59		
0,15	—	—		
0,50	—	—		
0,56	—	—		
0,22	—	—		
1,65	—	—		
0,14	—	—		
0,29	—	—		
—	0,65	0,22		
0,08	—	—		
0,19	0,60	0,21		
0,37	—	—		
0,83	—	—		
1,18	—	—		
0,13	—	—		
0,26	—	—		
0,24	—	—		
0,24	—	—		
0,32	—	—		
0,39	0,76	0,24		
—	1,78	0,98		
0,16	—	—		
0,23	—	—		
0,38	—	—		
0,12	—	—		
0,26	—	—		
1,65	—	—		
1,95	—	—		
0,25	—	—		
0,12	—	—		
0,23	—	—		
0,13	—	—		
0,08	—	—		
1,05	—	—		
0,10	—	—		
0,11	—	—		

0,25	—	—		
0,35	—	—		
0,53	—	—		
0,18	—	—		
0,09	—	—		
0,42	—	—		
0,14	—	—		
0,36	—	—		
1,93	—	—		
0,30	—	—		
0,19	—	—		
0,24	—	—		
0,26	—	—		
1,98	—	—		
0,63	—	—		
9,66	—	—		
0,48	—	—		
0,10	—	—		
0,10	—	—		
1,17	—	—		
2,97	—	—		
3,66	—	—		
0,32	—	—		
0,34	—	—		
1,24	—	—		
2,83	—	—		
2,00	2,41	1,21		
1,34	—	—		
2,41	—	—		
0,15	—	—		
0,57	—	—		
1,12	1,95	1,19		
—	1,47	0,48		
0,32	—	—		
0,56	—	—		
0,12	—	—		
0,62	—	—		
0,36	—	—		
0,05	—	—		
0,05	—	—		
0,34	—	—		
0,98	—	—		
0,26	—	—		
0,30	—	—		
0,18	—	—		
0,16	—	—		
1,28	—	—		
0,55	—	—		
0,12	—	—		
0,76	—	—		
0,46	—	—		
0,85	—	—		
0,05	—	—		
—	1,37	0,20		
0,25	—	—		
0,35	—	—		

0,62	1,80	0,53		
—	85,46	131,07		
0,64	1,83	1,08		
0,53	—	—		
0,22	—	—		
1,93	—	—		
0,67	—	—		
—	1,40	0,14		
0,15	—	—		
0,23	—	—		
—	1,54	0,31		
0,19	—	—		
0,10	—	—		
0,10	—	—		
0,16	—	—		
0,34	—	—		
0,58	—	—		
0,38	—	—		
0,19	—	—		
9,18	—	—		
0,15	—	—		
1,13	—	—		
0,21	—	—		
1,33	—	—		
1,39	—	—		
—	2,25	0,58		
0,15	—	—		
0,67	—	—		
0,82	—	—		
0,48	—	—		
0,93	—	—		
6,72	—	—		
0,19	—	—		
2,91	—	—		
0,05	—	—		
1,20	—	—		
1,60	—	—		
0,68	—	—		
—	1,59	0,66		
1,05	—	—		
0,18	—	—		
0,43	—	—		
0,16	—	—		
1,39	—	—		
0,11	—	—		
0,07	—	—		
0,33	—	—		
1,21	—	—		
—	1,41	0,23		
